# Dairy Product Consumption in Relation to Incident Prediabetes and Longitudinal Insulin Resistance in the Rotterdam Study

**DOI:** 10.3390/nu14030415

**Published:** 2022-01-18

**Authors:** Isabel A. L. Slurink, Trudy Voortman, Carolina Ochoa-Rosales, Fariba Ahmadizar, Maryam Kavousi, Nina Kupper, Tom Smeets, Sabita S. Soedamah-Muthu

**Affiliations:** 1 Center of Research on Psychological Disorders and Somatic Diseases (CORPS), Department of Medical and Clinical Psychology, Tilburg University, 5000 LE Tilburg, The Netherlands; H.M.Kupper@tilburguniversity.edu (N.K.); T.Smeets@tilburguniversity.edu (T.S.); S.S.Soedamah@tilburguniversity.edu (S.S.S.-M.); 2 Department of Epidemiology, Erasmus MC, University Medical Center Rotterdam, 3000 CA Rotterdam, The Netherlands; trudy.voortman@erasmusmc.nl (T.V.); C.Ochoarosales@erasmusmc.nl (C.O.-R.); F.Ahmadizar@umcutrecht.nl (F.A.); M.Kavousi@erasmusmc.nl (M.K.); 3 Division of Human Nutrition and Health, Wageningen University & Research, 6700 AA Wageningen, The Netherlands; 4 Centro de Vida Saludable, Universidad de Concepción, Concepción 4070374, Chile; 5 Julius Global Health, University Utrecht Medical Center, 3584 CG Utrecht, The Netherlands; 6 Institute for Food, Nutrition and Health, University of Reading, Reading RG6 6AR, UK

**Keywords:** dairy, milk, yogurt, impaired glucose metabolism, impaired fasting glucose, prediabetes, insulin resistance, type 2 diabetes, prospective cohort

## Abstract

Evidence suggests neutral or moderately beneficial effects of dairy intake on type 2 diabetes mellitus risk. Nevertheless, evidence on associations with early phases of type 2 diabetes remains inconsistent. We aimed to examine associations between dairy-type intake with prediabetes risk and longitudinal insulin resistance. The analytic sample consisted of 6770 participants (aged 62 ± 4 years, 59% female) free of (pre-)diabetes at baseline from the prospective population-based Rotterdam Study. Dairy intake was measured at baseline using food frequency questionnaires. Data on prediabetes (fasting blood glucose 6.1–6.9 mmol/L or non-fasting 7.7–11.1 mmol/L) and the longitudinal homeostatic model assessment of insulin resistance (HOMA-IR) were available from 1993–2015. Associations with these outcomes were analyzed with dairy intake in quartiles (Q4 vs. Q1) and continuous using multivariable Cox proportional hazard models and linear mixed models. During a mean follow-up of 11.3 ± 4.8 years, 1139 incident prediabetes cases were documented (18.8%). In models adjusting for sociodemographic, lifestyle and dietary factors, a higher intake of high-fat yogurt was associated with lower prediabetes risk (HR_Q4vsQ1_ 0.70, 95% CI 0.54–0.91 and HR_serving/day_ 0.67, 0.51–0.89). In addition, a higher intake of high-fat milk was associated with lower prediabetes risk (HR_Q4vsQ1_ 0.81, 0.67–0.97, HR_serving/day_ 0.88, 0.79–0.99). Associations were found for low-fat dairy, low-fat milk and total cheese with a higher prediabetes risk (HR_serving/day_ ranging from 1.05–1.07, not significant in quartiles). Associations with longitudinal HOMA-IR were similar to prediabetes for high-fat yogurt, low-fat dairy and low-fat milk. Fermented dairy, low-fat yogurt, high-fat cheese, cream and ice cream were not associated with the outcomes. In conclusion, a higher intake of high-fat yogurt was associated with a lower prediabetes risk and lower longitudinal insulin resistance. Additionally, high-fat milk was associated with a lower prediabetes risk. Some low-fat dairy types were inconsistently associated with these outcomes. Studies are needed to confirm associations and to examine the influence of confounding by population characteristics.

## 1. Introduction

The diabetes mellitus epidemic is a global public health problem, making it crucial to identify preventive strategies effective in the early stages of the disease. In the early stages, progressive loss of β-cell capacity and mass result in the development of insulin resistance and elevation of fasting blood glucose [1]. Prediabetes is a widely prevalent condition, characterized by elevated blood glucose levels above the normal range, but below the diagnostic threshold for type 2 diabetes mellitus [2]. Prediabetes is associated with cardiovascular disease and mortality [3,4] and likely causally with coronary artery disease, as concluded by a Mendelian randomization study [3]. Thus, it is essential to identify modifiable risk factors that could prevent or revert insulin resistance and prediabetes or delay its transitions to type 2 diabetes.

An unhealthy diet, physical inactivity and excess weight are of major importance in the development of type 2 diabetes, and many cases can be prevented with lifestyle modifications [5,6,7,8]. Dairy products can be an interesting preventive target in maintaining cardiometabolic health, as they are a rich source of calcium, potassium and vitamins. Furthermore, dairy proteins have been associated with favorable body composition and improved insulin sensitivity [9]. Vitamin K2 (menaquinones) may improve insulin sensitivity via several pathways [10] and has been associated with reduced type 2 diabetes risk [11]. Nevertheless, dairy also contains sodium, saturated fatty acids (SFAs) and may contain added sugars. Dietary guidelines of many countries worldwide recommend consuming 1 to 4 servings of dairy foods daily, focusing on selecting low-fat options to lower SFA intake [12]. However, this recommendation is insufficiently substantiated as evidence on the harmful effects of high-fat dairy on cardiometabolic health is lacking [13,14]. The health effects differ by individual SFA type, further modulated by the dairy food matrix [9]. Thus, there has been controversy concerning the place of dairy and its subtypes in healthy diets [15].

The overall evidence indicates a neutral or moderately beneficial association between dairy intake and type 2 diabetes, especially yogurt [16,17,18]. Not much is known about dairy in relation to earlier phases of type 2 diabetes, while this may provide further insights into its role in the etiology of the disease. Only two prospective cohort studies have studied the associations of dairy and prediabetes risk. Data from The US Framingham Offspring Cohort with 12 years of follow-up (*n* = 1867) showed a 39%, 32% and 25% lower prediabetes risk for, respectively, total, low-fat and high-fat dairy for the top vs. bottom quartile and nonlinear inverse associations for milk and yogurt [19]. On the contrary, in our recent analysis in the Dutch Hoorn studies (*n* = 2262; [20]), we did not observe associations for total dairy or most of the studied dairy types and prediabetes risk. However, high-fat fermented dairy, cheese and high-fat cheese were associated with a lower risk of prediabetes during a mean follow-up of 6.4 years. In the cross-sectional ELSA-Brasil study, inverse associations of dairy and insulin resistance were found [21]. A meta-analysis from Randomized Controlled Trials (RCTs) showed that beneficial effects of dairy on insulin resistance were more likely to be observed in studies longer than 12 weeks [22]. Nevertheless, studies with long-term follow-up to confirm these results are lacking. Considerable heterogeneity between results, possibly explained by variation in dairy type intake or outcome definition, underline the need for extensive longitudinal studies.

Therefore, we examined associations between the consumption of total dairy and dairy subtypes with incident prediabetes and longitudinal insulin resistance in the prospective Rotterdam Study populations.

## 2. Materials and Methods

### 2.1. Study Population

This study was embedded in three sub-cohorts of the Rotterdam Study (RS), a prospective cohort study ongoing since 1990. It is comprised of middle-aged and elderly persons living in the district Ommoord in Rotterdam, the Netherlands. Details of the study design are described elsewhere [23]. The first sub-cohort (RS-I) was established in 1989–1993 among inhabitants aged 55 and over (*n* = 7983). The second sub-cohort (RS-II) was recruited in January 2000 among people who had become 55 years of age or moved into the study district (*n* = 3011). The third sub-cohort (RS-III) was initiated in 2006 for which subjects aged 45 years and older were recruited (*n* = 3932). These three sub-cohorts of the Rotterdam Study comprised of 14926 subjects at baseline with an overall response of 72%. Examinations were repeated every 3–5 years. The study was approved by the Medical Ethics Committee of Erasmus University Medical Centre, and all participants provided written informed consent.

For the current analysis, we excluded participants with prevalent type 2 diabetes and without dietary data at baseline, resulting in 6770 participants (RS-I-1: *n* = 2971, RS-II-1: *n* = 1413, and RS-III-1: *n* = 2386) (Figure S1). For the prediabetes incidence analysis, we additionally excluded participants with prediabetes at baseline or without follow-up data on prediabetes, resulting in 6053 participants (RS-I: *n* = 2617, RS-II: *n* = 1250, RS-III: *n* = 2186). For analyses on insulin resistance, we excluded participants without data on the homeostatic model assessment of insulin resistance (HOMA-IR) at baseline and follow-up, resulting in 6593 participants (RS-I: *n* = 2892, RS-II: *n* = 1391, RS-III: *n* = 2310). Data on outcome measures were available until 2015.

### 2.2. Assessment of Dairy Intake

Food intake data at baseline and follow-up were obtained with a 170-item (RS-I-1 and RS-II-1) and with a 389-item (RS-III-1, RS-I-5 and RS-II-3) food frequency questionnaire (FFQ) as described in detail elsewhere [24]. The FFQs were checked during an interview by a trained dietician at the study center. The 170-item FFQ was validated in 80 RS participants against fifteen 24 h food records with adjusted Pearson’s correlations of 0.66 for protein intake, 0.52 for saturated fat intake, 0.58 for sodium intake and against 24 h urine collections showing a Spearman correlation coefficient of 0.67 for protein [25]. The 389-item FFQ was validated in two Dutch populations against a 9 day dietary record and a 4 week dietary history with Pearson’s correlation coefficients of 0.61 for total protein intake, 0.73–0.75 for saturated fat and 0.60 for calcium, and 0.60 for milk and milk products and 0.61 for cheese [26,27]. Nutrient and energy intake were calculated using the Dutch Food Composition Tables 1993, 2001, 2006 and 2011 (NEVO) depending on the year of data collection in the sub-cohorts. Participants with an unreliable dietary intake according to the trained dietician or extreme energy intakes (<500 or >5000 kcal/day) were excluded [24]. Dairy categories included total dairy, fermented dairy, milk, yogurt, cheese, cream and ice cream (Table S1). Each dairy category was further divided into low-fat (liquid products ≤ 2%, cheese ≤ 20%) and high-fat (liquid products > 2%, cheese > 20%). Intakes were expressed in servings/day according to Dutch serving sizes: milk, 200 mL; yogurt, 150 mL; cheese, 20 g; cream 3 g; ice cream, 50 g (https://portie-online.rivm.nl/, accessed on 1 September 2021). In the total dairy category, a serving of liquid dairy products was defined as 200 mL and cheese as 20 g.

### 2.3. Assessment of Outcomes

Fasting blood was drawn at two time points in each sub-cohort; at RS-I-3 (1997–1999) and I-5 (2009–2011), at RS-II-1 (2000–2001) and II-3 (2011–2012), and RS-III-1 (2006–2008) and III-2 (2012–2014). Glucose levels were measured using the glucose hexokinase method [28]. We set the third visit of RS-I (RS-I-3; 1997–1999) as a baseline, as fasting blood samples were not collected at the first two visits of RS-I. Information from general practitioners, structured home interviews, pharmacy dispensing records and follow-up examinations in the research facility was used to identify prediabetes and type 2 diabetes cases at baseline and during follow-up. Prediabetes was defined as having a fasting blood glucose between 6.1 and 6.9 mmol/L or non-fasting blood glucose between 7.7 and 11.1 mmol/L, according to WHO guidelines [2]. Type 2 diabetes was defined as a fasting plasma glucose level ≥ 7 mmol/L, a non-fasting plasma glucose level ≥ 11.1 mmol/L or the use of blood glucose-lowering medication [2]. All cases were independently identified by two study physicians. In case of a disagreement, a consensus was sought by consulting endocrinologists. Serum insulin levels were measured using the Roche Modular Analytics E170 analyzer. The HOMA-IR was calculated by multiplying fasting insulin (mU/L) by fasting glucose (mmol/L) divided by 22.5.

### 2.4. Assessment of Covariates

Information on demographic factors, education, health status, medical history and smoking behavior was obtained during home interviews at baseline. Education attainment was defined as primary (primary education), low (lower/intermediate general education or lower vocational education), intermediate (intermediate vocational education or higher general education) or high (higher vocational education or university). Participants were classified as never, former or current smokers. Height (cm) and weight (kg) were assessed during a physical examination at the research center, and body mass index (BMI) was calculated as kg/m^2^. Waist circumference (WC) was assessed at baseline and during follow-up (RS-I-3 (1997–1999), RS-I-4 (2002–2004), RS-I-5 (2009–2011) and RS-I-6 (2014–2015); RS-II-2 (2004–2005), RS-II-3 (2011–2012) and RS-II-4 (2015–2016); and RS-III-2 (2012–2014)). WC in cm was measured at the level midway between the lower rib margin and the iliac crest with the participant in a standing position. Data on physical activity (PA) expressed in metabolic equivalent of task (MET) minutes per week were obtained using the Zutphen Physical Activity Questionnaire for RS-I-3 and RS-II-1 [29] and using the LASA Physical Activity Questionnaire (LAPAQ) for RS-III-1 [30,31]. Diet quality was expressed as adherence to 14 food groups of the Dutch Dietary Guidelines 2015 [24,32]. Information on medication use was obtained from both home interviews and pharmacy dispensing records. Blood pressure was calculated as the mean of two consecutive measurements with a random-zero sphygmomanometer while subjects were in a sitting position and had rested for 5 min. Hypertension was defined as: systolic blood pressure ≥ 140 mmHg; and/or diastolic blood pressure ≥ 90 mmHg; and/or use of antihypertensive medication. Serum total cholesterol and HDL cholesterol were measured with the use of an automatic enzymatic procedure. Information on family history of diabetes was available at RS-I-1 and RS-II-1 and was defined as having at least one parent or sibling with type 2 diabetes. Coronary heart disease (CHD) at baseline was defined as having a medical record of myocardial infarction and at follow-up as myocardial infarction or definite coronary mortality [33].

### 2.5. Statistical Analysis

Descriptive data were presented as means and standard deviations (SD) for continuous variables, medians and interquartile ranges (IQR) for non-normally distributed continuous variables and frequencies and percentages for categorical variables. Natural log-transformed values for HOMA-IR were used to approximate normal data distributions.

To analyze associations between the various dairy types and prediabetes incidence, we used Cox proportional hazard models. Results were expressed as a Hazard Ratio and 95% confidence intervals (HR, 95% CI). To analyze associations between dairy types and longitudinal HOMA-IR, we performed linear mixed models with time as a fixed effect, a random intercept for participants and a random slope for the time of repeated HOMA-IR measures. Results were expressed as beta coefficients of log-transformed HOMA-IR and 95% confidence intervals (β, 95% CI). Dairy types were analyzed as categorical variables based on quartiles, comparing the highest versus lowest (reference) quartile of intake, and continuous variables (servings/day). Dairy types for which many participants reported no intake were categorized into a non-consumer category (reference) and consumers into tertiles. The *p* for the trend was calculated using the median values of dairy intake range categories as continuous variables in the model. For each model, we examined whether non-linear terms of continuous dairy types (2nd order polynomials or natural splines with 3 degrees of freedom, excluding outliers) significantly improved model fit compared to the linear model assessed by likelihood ratio tests.

Confounders were chosen based on previous research [32,34,35]. The basic model (model 1) was adjusted for age, sex and daily energy intake. Model 2 was additionally adjusted for educational attainment, alcohol intake, smoking status, physical activity, family history of type 2 diabetes (RS-I and RS-II only), intake of fruits, vegetables, whole grains, legumes, nuts, tea, coffee, red meat and sugar-sweetened beverages (SSB). We presented descriptive data stratified by the dairy types significantly associated with the outcomes to provide insight in characteristics of participants with high and low intakes. Effect modification by age, sex and WC were examined in model 2, and stratified associations were presented in case of significant interactions (*p* < 0.05).

Multiple sensitivity analyses were performed in model 2 to examine the robustness of the findings. First, we additionally adjusted for longitudinal WC to examine the potential confounding or mediating effect of obesity over time in associations of dairy, prediabetes incidence and longitudinal HOMA-IR. To adjust for longitudinal WC in models of prediabetes incidence, we applied a joint modelling approach [36]. With this approach, model 2 was combined with a random slope linear mixed model, including the repeated measures of WC before the onset of prediabetes as an outcome, time of WC measurements and interactions between dairy types and time of WC measurements. Second, we additionally adjusted for cholesterol, hypertension and triglycerides, as these factors are potential mediators. Third, we additionally adjusted for consumption of other dairy types to assess whether associations of certain dairy types were independent of each other. Fourth, participants with prevalent or incident CHD were excluded to address reverse causation by the change of diet and lifestyle. Fifth, associations were calculated with energy-adjusted intake of dairy types in gram/day using the residual method. Sixth, repeated measures of dairy intake (baseline and measures 20 years after baseline in RS-I (*n* = 1028, 34.6%) and 10 years after baseline in RS-II (*n* = 859, 60.8%)) were included in Cox models as time-dependent exposure and in linear mixed models as a fixed effect. Note that for most participants, the Cox model used baseline measures of dairy only because most prediabetes cases occurred before the repeated dietary assessment. Therefore, we additionally adjusted for dairy intake at follow-up in the subset with these data available to explore potential effects of altered dairy intake.

All analyses were performed separately for RS-I, RS-II and RS-III, and the results were pooled using a fixed-effects meta-analysis. To adjust for potential bias associated with missing data, a multiple imputation procedure (*n* = 10) was used to account for missing data on covariates (Table S2). No correction for multiple testing was made, as most exposures were correlated and corrections may have resulted in a type II error [37]. Statistical procedures were performed using SPSS statistical software, version 21.0 (IBM Corp, Armonk, NY, USA) and R version 4.0.2. (The R Foundation for Statistical Computing, Vienna, Austria).

## 3. Results

### 3.1. Population Characteristics

**Figure 1:**
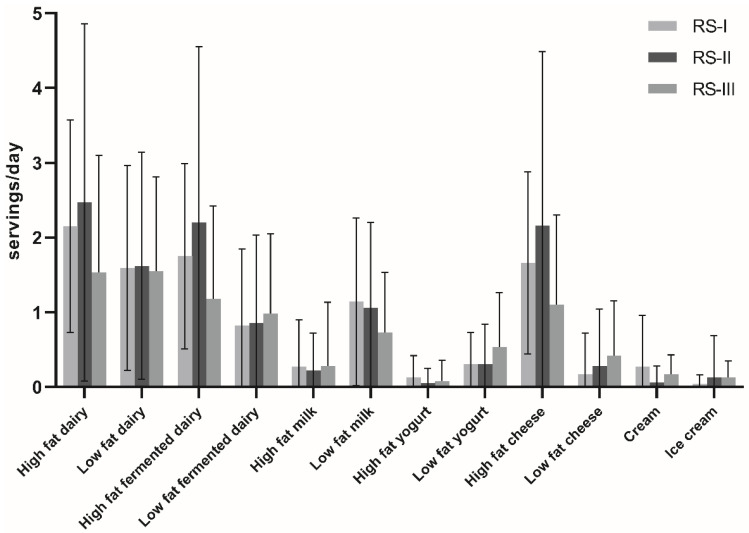
Dairy-type intake by cohort of the Rotterdam Studies in servings/day (mean ± SD): milk, 200 mL; yogurt, 150 mL; cheese, 20 g; cream 3 g; ice cream, 50 g. Combined total dairy category: liquid dairy products, 200 mL; cheese, 20 g. RS, Rotterdam Study.

The mean total dairy intake was 3.6 ± 1.2 servings/day, mostly consisting of low-fat milk (0.9 ± 0.6) and high-fat cheese consumption (1.5 ± 0.8) (Figure 1). The mean age was 61.7 ± 3.9 years, and 58.7% were female (Table 1). The mean waist circumference was 91.1 ± 6.7 cm, the mean BMI was 26.6 ± 2.2 kg/m^2^ and 16% were obese (BMI ≥ 30 kg/m^2^). In the highest (6.0 ± 1.1 servings/day) compared to the lowest quartile of dairy intake (1.5 ± 0.3 servings/day), participants were more often highly educated (21.4 vs. 16.1%) and less often smokers (22.4 vs. 27.0%). Furthermore, diet quality, energy intake and intakes of vegetables, fruit, whole grains, sodium and calcium were on average higher with increasing dairy intake. Characteristics by intake of specific dairy types are presented in Table S3. Stratified by cohort, RS-III was on average younger (56.8 ± 6.4, 65.5 ± 6.7, 63.6 ± 7.2 years in RS-III, -I, -II, respectively), with more highly educated participants (28.7% vs. 10.1 and 18.1%) (Table S4). Participants included in this study (*n* = 6770) compared to those who were excluded (*n* = 8162) were generally younger (62.0 ± 7.8 vs. 69.2 ± 11.4), higher educated (18.3 vs. 11.6%), less often smokers (22.7 vs. 25.6%), had higher physical activity levels and lower HOMA-IR levels (2.9 ± 2.4 vs. 5.6 ± 13.0) (Table S5).

### 3.2. Dairy Intake and Prediabetes Risk

During a mean follow-up of 11.4 ± 4.8 years, 1139 incident prediabetes cases were identified among 6053 participants (18.8%). In pooled multivariable models (Table 2, model 2), high-fat yogurt (19% of sum total yogurt consumption) was associated with a lower prediabetes risk (HR_Q4vsQ1_ 0.70, 95% CI 0.54–0.91 and HR_serving/day_ 0.67, 95% CI 0.51–0.89). Additionally, high-fat milk (21% of total milk) was associated with a lower prediabetes risk (HR_Q4vsQ1_ 0.81, 0.67–0.97 and HR_serving/day_ 0.88, 95% CI 0.79–0.99). In contrast, low-fat dairy, low-fat milk and total cheese were associated with a higher prediabetes risk when analyzed on a continuous scale and not in quartiles (HR_serving/day_, respectively, 1.05, 1.01–1.10; 1.07, 1.01–1.13; 1.05, 1.01–1.09). In addition, low-fat cheese was associated with a higher prediabetes risk when analyzed in quartiles (15% of total cheese, HR_Q4vsQ1_ 1.17, 95% CI 0.95–1.44, p_trend_ = 0.04). Total and high-fat dairy; total, high-fat and low-fat fermented dairy; total milk; total and low-fat yogurt; high-fat cheese; cream and ice cream were not associated with a prediabetes risk.

### 3.3. Dairy Intake and Longitudinal Insulin Resistance

The median HOMA-IR index was 2.3 (IQR 1.7–3.4) at baseline and 2.4 (1.7–3.8) at follow-up. In line with results for prediabetes, high-fat yogurt was associated with lower longitudinal log-transformed HOMA-IR (β_Q4vsQ1_ −0.10, 95% CI −0.16, −0.05, p_trend_ = 0.0003, β_serving/day_ −0.08, 95% CI −0.13, −0.03) (Table 3, model 2). Low-fat dairy and low-fat milk were associated with higher longitudinal log-HOMA-IR (β_Q4vsQ1_, respectively, 0.06, 95% CI 0.03–0.10, p_trend_ = 0.0003 and 0.07, 0.03–0.11, p_trend_ = 0.001, and β_serving/day_, respectively, 0.02, 0.01–0.03 and 0.02, 0.01–0.04). Total milk was significantly associated with higher longitudinal log-HOMA-IR only when comparing top vs. bottom quartiles (β_Q4vsQ1_ 0.05, 0.01–0.09, p_trend_ = 0.02). In contrast to observations for prediabetes risk, total dairy, high-fat milk, and total and low-fat cheese were not associated with longitudinal HOMA-IR. A better fit of non-linear associations was found for low-fat fermented dairy, low-fat milk, high-fat milk, low-fat yogurt, and cream, but non-linear trends were inconclusive, as only a few participants had high intakes (Figure S2).

### 3.4. Associations in Sub-Cohorts

The association of a higher intake of high-fat yogurt with a lower prediabetes risk and longitudinal insulin resistance was consistently found in all three sub-cohorts (Tables S6 and S7). For other dairy types, some discrepancies were found. Associations of low-fat dairy and low-fat milk with a higher prediabetes risk were found in RS-I and RS-II but not in RS-III, whereas the positive associations with HOMA-IR were observed in RS-II and RS-III but not in RS-I. The positive association for total cheese and prediabetes was observed in RS-I but not in RS-II and RS-III.

### 3.5. Sensitivity Analysis

We observed significant interactions of dairy consumption with sex and WC on prediabetes risk (Table S8) and with sex, age and WC on HOMA-IR (Table S9). However, stratified analyses revealed no clear patterns. Associations of dairy types with prediabetes and HOMA-IR were comparable, although for most, no longer statistically significant after additionally adjusting for longitudinal WC (model 3) (Tables S10 and S11). Furthermore, all associations were similar or only slightly attenuated after additional mutual adjustment for other dairy types; additional adjustment for cholesterol, hypertension and triglycerides; exclusion of participants with prevalent and incident CHD; or using dairy intake as an energy adjusted variable with the residual method, instead. In a subsample of RS-I and RS-II with repeated dietary intake assessment, dairy intake at follow-up was similar to baseline—only total cheese intake was higher in RS-I (2.5 ± 2.1 vs. 1.9 ± 1.1 servings/day) (Table S12). After adjustment for dairy intake at follow-up, associations generally remained similar, except for low-fat dairy, total milk and low-fat milk in RS-II (Tables S13 and S14).

## 4. Discussion

In this population-based cohort study, high-fat yogurt was consistently associated with a lower prediabetes risk and lower longitudinal insulin resistance. Additionally, high-fat milk was associated with a lower prediabetes risk but not with longitudinal insulin resistance. Higher intakes of low-fat dairy, low-fat milk and total and low-fat cheese were associated with a higher prediabetes risk but inconsistently across sub-cohorts and by variable type (continuous or quartiles). Higher intake of low-fat dairy and total and low-fat milk were associated with a higher longitudinal insulin resistance. Total dairy, fermented dairy, low-fat yogurt, high-fat cheese, cream and ice cream were not associated with prediabetes risk or longitudinal insulin resistance.

Of the dairy types examined in our study, high-fat yogurt intake was most strongly associated with prediabetes and insulin resistance, consistent across all three sub-cohorts and robust in sensitivity analyses. Generally, prior studies lack information on the fat content of yogurt. Previous meta-analyses of observational studies showed that a higher compared to a lower intake of total yogurt is significantly associated with a lower type 2 diabetes risk (relative risks (RRs) ranging from 0.74–0.86 in five meta-analyses) [16,17]. There are limited prospective [19,20] and cross-sectional studies with prediabetes as the specific outcome [38,39]. In the FHS Offspring cohort, yogurt intake was non-linearly associated with prediabetes, with the lowest risk at 2–4 servings/week but an increased risk with higher intakes [19]. In addition, the Dutch Maastricht cohort (*n* = 3451) showed that the highest vs. the lowest intake of yogurt was associated with lower odds of prediabetes (OR 0.67, 95% CI 0.50–0.90) [38]. On the contrary, no associations of yogurt with prediabetes were found in two other Dutch studies: the prospective Hoorn studies [20] and Lifelines study [39]. An important explanation for heterogeneous associations between populations may be the potential confounding by unmeasured differences in population characteristics and health status. In Dutch populations, dairy foods are consumed by various population groups and within a wide range of diets, while, for example in the US, high dairy intake generally reflects overall healthier behavior [40]. Furthermore, the quantity and composition of dairy-type categories vary by the availability of products and consumption habits in a region. FFQs do not assess the sugar content of yogurt products. The intake of plain and sugar-sweetened yogurt may differ between populations, both plausibly differentially associated with cardio metabolic outcomes. Overall, the evidence indicates a neutral or inverse association between yogurt and early phases of type 2 diabetes, yet there is a need for studies explaining heterogeneity and further examining the role of fat content on cardio metabolic effects.

In our cohort, a higher intake of high-fat milk was associated with a lower prediabetes risk, but not with longitudinal insulin resistance. In the FHS Offspring Cohort, a non-linear association was found for high-fat milk, with moderate intakes associated with a lower prediabetes risk but a higher risk with higher intakes [19]. No associations with high-fat milk or milk were found in the prospective Hoorn Study [20] and two Dutch cross-sectional studies [38,39]. Meta-analyses of observational studies indicate neutral associations of milk and type 2 diabetes, confirmed by Mendelian randomization studies [41].

The associations found for higher intake of total dairy, low-fat dairy, low-fat milk and total and low-fat cheese and the outcomes were somewhat weaker and not found in the most recent Rotterdam sub-cohort. Previous analyses of the Rotterdam study showed that protein from dairy was associated with a higher prediabetes risk (HR per 5% energy increment 1.26, 95% CI 1.06–1.49) and longitudinal insulin resistance (β 0.04, 95% CI 0.0003, 0.08), independent of other macronutrients and diet quality [42]. This suggests that specifically dairy protein intake might underlie the positive associations between low-fat dairy types and outcomes in the Rotterdam Study. Our results differ from the previous evidence of prospective studies [19,20]. The FHS Offspring Cohort (*n* = 1867) reported beneficial associations for total and low-fat dairy [19], and the Dutch Hoorn study (*n* = 2262) found no associations between low-fat dairy types and prediabetes risk [20]. The intake of low-fat dairy and total protein was slightly higher in the Rotterdam Study compared to the Hoorn study, plausibly explaining different associations. In addition, with higher low-fat dairy intake, energy intake and total carbohydrate intake was also higher, yet associations were independent of major sources of carbohydrates, suggesting that protein content could be responsible for the associations. These associations need further confirmation in trials. So far, the evidence from trials is inconclusive. A recent meta-analysis from our group of 54 controlled dietary intervention studies reported that higher protein diets led to greater weight loss, fat mass loss and beneficial reductions in systolic blood pressure and improved lipid and insulin outcomes compared to lower protein diets over a follow-up period of four to five months [43]. This study showed no detrimental effects and some beneficial effects of higher protein diets on body weight and markers of cardiometabolic health. Longer-term trials are warranted to give insights on the effects of specific dairy proteins [44]. Furthermore, our results contradict with meta-analyses of observational studies showing mostly neutral or slightly beneficial associations between these dairy types and type 2 diabetes [16].

An additional source of inconsistencies in the associations of dairy types with prediabetes and insulin resistance may arise from the inclusion of participants with various metabolic states at baseline [19]. Potential non-linear associations and less pronounced effect estimates in insulin resistance models and potential measurement error warrant replication studies. Recent meta-analyses of studies using experimental designs show contradicting results [45,46]. Null associations (13 RCTS, *n* = 840) [45] as well as significant reductions in HOMA-IR (14 RCTs, *n* = 794) [46] have been reported when comparing diets high and low in dairy. In addition, a recent RCT showed that both high-fat and low-fat dairy diets, compared to a diet low in dairy, were associated with decreased insulin sensitivity [47]. Nevertheless, it is worthy to note that this study included participants with the metabolic syndrome, and the primary outcome, glucose tolerance, did not change. Overall, these interventions were heterogeneous in the study population (age, co-morbidities), duration and treatment and control diets. Furthermore, the authors did not specify the dairy type or fat content. It is unknown if these results translate to long-term risk of diabetes, for which future well-designed trials and long-term studies are needed.

Multiple explanations have been proposed linking dairy intake, especially yogurt, to a lower risk of type 2 diabetes development, although causal molecular mechanisms remain unclear [9]. Yogurt contains probiotics originating from the fermentation process. Probiotics have been associated with lower weight gain, lower cholesterol and blood glucose levels in animal models, possibly by compositional and functional changes in the gut microbiome, increased butyrate production and anti-inflammatory effects [9,44,48]. In a study in people with prediabetes, participants with daily intake of yogurt enriched with *Lactobacillus plantarum* showed greater reductions in HbA1c levels compared to participants with a daily intake of conventional yogurt [49]. Some dairy fats and proteins have been related to pathways linked to a lower risk of prediabetes in animal and in vitro studies, albeit the content of these specific nutrients in yogurt is low. For example, branched-chain and ruminant trans fatty acids may inhibit hepatic de novo lipogenesis, improve insulin resistance and reduce inflammation [9,50]. In vitro, whey proteins have shown to upregulate hepatic glucose metabolism through gene expression regulation [51]. Branched-chain amino acids may activate the mammalian target of the rapamycin complex (mTOR) signaling pathway upregulating insulin secretion, resulting in enhanced glucose clearance [52]. Yet, prolonged increased insulin levels may lead to insulin resistance and type 2 diabetes [53]. Nevertheless, high protein diets show favorable effects on weight loss in RCTs [43], related to effects on gut-derived hormones and thermogenesis promoting satiating, and preservation of fat-free mass during weight loss [54]. We did observe that both beneficial associations of high-fat yogurt and positive associations of low-fat dairy slightly attenuated after adjustment for longitudinal WC, suggesting obesity may partly mediate some associations [42]. However, we observed that associations were independent of blood lipids and hypertension, suggesting that these factors did not play a role.

The current study has multiple strengths. First, we examined associations of several dairy types with both prediabetes and insulin resistance in a large population-based cohort. In addition, our study provided temporal associations with repeated measures of insulin resistance and a considerable follow-up duration. Second, to our knowledge, this is the first study examining associations of dairy with repeated measures of insulin resistance. Third, the associations were controlled for a wide range of confounders. These included major energy-providing food groups previously associated with development of type 2 diabetes to prevent confounding by background diet, which is not widely done in dairy-diabetes research, thus improving the quality of the current evidence on dairy-diabetes research [55].

There are also some limitations to the current study. First, measurement errors in the habitual dairy intake assessment, for example due to recall bias, may result in bias towards the null. Furthermore, dairy intake might have changed over time. However, a sensitivity analysis incorporating repeated measures of dairy consumption and excluding participants likely to change their diet due to diagnosis of cardiovascular diseases showed similar associations for most dairy types. Second, the between-person variation in the intake of several dairy products, such as high-fat yogurt and low-fat cheese, were limited due to the observational nature of our study. Third, residual and unmeasured confounding can never be ruled out in observational studies, for example, by potential effects of meal frequency and timing and replacement choices for dairy consumption [56,57]. Fourth, no 2 h plasma glucose levels were available, and using the FPG only to define prediabetes cases may lead to underestimation of prediabetes cases [2]. This possible non-differential misclassification of the outcome may have resulted in bias towards the null.

## 5. Conclusions

In this population-based cohort study, high-fat yogurt showed robust inverse associations with a prediabetes risk and longitudinal insulin resistance. Higher intake of high-fat milk was also associated with a lower prediabetes risk. Low-fat dairy, total milk, low-fat milk and total and low-fat cheese were positively associated with the outcomes but inconsistently. With the current study, we extend the understanding of the role of dairy intake before clinical stages and decreasing the risk of reverse causation by the presence of disease. Well-designed prospective cohort studies and long-term trials are needed to confirm associations and to explore confounding factors.

## Figures and Tables

**Table 1:** Baseline characteristics of study population (*n* = 6770) according to quartiles of total dairy intake.

	Total	Q1	Q2	Q3	Q4
	*n* = 6770	*n* = 1692	*n* = 1697	*n* = 1688	*n* = 1693
Total dairy intake (servings/day)	3.6 ± 1.2	1.5 ± 0.3	2.8 ± 0.2	3.9 ± 0.2	6.0 ± 1.1
Range	0–15.1	0–1.2	0.9–1.9	1.4–2.7	2.2–15.1
Age at dietary assessment (years)	61.7 ± 3.9	62.1 ± 4.0	62.0 ± 3.9	61.8 ± 3.8	60.9 ± 3.7
Sex, female (%)	58.7	59.9	61.7	59.6	53.8
BMI (kg/m^2^)	26.6 ± 2.2	26.7 ± 2.2	26.7 ± 2.2	26.6 ± 2.2	26.6 ± 2.2
Waist circumference (cm)	91.1 ± 6.7	91.1 ± 6.7	90.7 ± 6.6	91.0 ± 6.5	91.6 ± 6.8
Education level (%)					
Primary education	11.8	13.7	11.2	11.4	11.0
Lower education	40.9	42.2	43.8	40.1	37.6
Intermediate	28.9	28.0	28.0	29.7	30.0
Higher	18.3	16.1	17.0	18.8	21.4
Smoking (%)					
Never	32.2	31.3	33.5	32.9	31.3
Ever	45.0	41.7	45.9	46.3	46.3
Current	22.7	27.0	20.6	20.8	22.4
Physical activity (MET-hours/week)					
Zutphen Physical Activity Questionnaire (*n* = 4328)	79.7 [54.7, 112.1]	77.7 [51.5, 110.8]	78.2 [53.0, 109.2]	79.4 [57.5, 111.9]	82.4 [56.6, 115.6]
LASA Physical Activity Questionnaire (*n* = 2177)	42.9 [17.7, 82.5]	39.8 [15.0, 75.9]	40.5 [16.9, 79.9]	48.2 [21.0, 87.8]	45.0 [18.0, 86.4]
Family history diabetes mellitus	12.8	12.7	12.6	12.6	13.3
*Dietary intake*					
Diet quality score (0–14)	6.6 ± 1.1	6.0 ± 1.0	6.6 ± 1.0	7.0 ± 1.1	7.0 ± 1.1
Energy intake (kcal/day)	2113 ± 333	1858 ± 293	2012 ± 283	2151 ± 285	2452 ± 365
Total fat intake (E%)	35.1 ± 3.6	35.3 ± 4.1	35.0 ± 3.5	34.5 ± 3.3	35.4 ± 3.6
Total saturated fat intake (E%)	13.2 ± 1.6	12.4 ± 1.6	12.9 ± 1.5	13.1 ± 1.4	14.1 ± 1.8
Total protein intake (E%)	16.7 ± 1.7	15.8 ± 1.6	16.4 ± 1.6	16.8 ± 1.5	17.6 ± 1.7
Carbohydrate intake (E%)	44.5 ± 4.2	44.5 ± 4.9	44.9 ± 4.1	44.9 ± 3.8	43.8 ± 3.9
Calcium intake (mg/day)	1109 ± 251	688 ± 113	960 ± 95	1175 ± 101	1621 ± 245
Sodium intake (mg/day)	2344 ± 463	1979 ± 385	2203 ± 366	2398 ± 372	2814 ± 511
Alcohol intake (g/day)	6.6 [0.7, 18.8]	6.7 [0.5, 20.9]	6.6 [0.7, 18.6]	6.2 [0.7, 17.6]	6.7 [0.7, 17.6]
Vegetables (g/day)	211 ± 69	206 ± 62	208 ± 71	207 ± 59	220 ± 74
Fruit (g/day)	228 ± 98	209 ± 102	230 ± 93	235 ± 94	234 ± 101
Wholegrains (g/day)	116 ± 43	95 ± 41	111 ± 41	123 ± 42	133 ± 46
Legumes (g/day)	16.5 ± 12.5	15.7 ± 14.6	16.4 ± 11.2	15.4 ± 9.9	17.5 ± 11.8
Nuts (g/day)	8.5 ± 7.9	7.9 ± 8.0	8.3 ± 7.9	8.5 ± 7.3	9.1 ± 8.1
Red meat (g/day)	93 ± 36	91 ± 35	92 ± 34	93 ± 32	97 ± 40
Fish (g/day)	20 ± 13	19 ± 13	21 ± 12	20 ± 13	21 ± 13
Tea (g/day)	288 ± 155	286 ± 162	275 ± 146	304 ± 152	286 ± 159
Coffee (g/day)	471 ± 152	445 ± 155	462 ± 144	475 ± 146	502 ± 159
Sugar sweetened beverages (g/day)	94 ± 74	92 ± 78	96 ± 74	86 ± 63	101 ± 79

Values are mean ± SD for continuous variables with a normal distribution (pooled), or median [IQR] for continuous variables with a skewed distribution; percentages for categorical variables, on the basis of unimputed data. Abbreviations: E%, percentage of total energy intake; MET, metabolic equivalent of task; RS, Rotterdam Study; SD, standard deviation.

**Table 2:** Associations of dairy product types and prediabetes risk in the Rotterdam Studies (*n* = 6053).

	Pooled Effect Estimates					
	Intake Range Categories					Continuous
	Q1	Q2	Q3	Q4		
	HR	HR (95% CI)	HR (95% CI)	HR (95% CI)	P_trend_	HR (95% CI)
**Total dairy**						
*n*/N	297/1512	287/1517	257/1513	298/1511		1139/6053
Median intake	1.6	2.8	3.9	5.6		3.3
Model 1	1 (ref)	0.96 (0.81–1.13)	0.85 (0.71–1.00)	1.00 (0.84–1.19)	0.89	1.02 (0.99–1.05)
Model 2	1 (ref)	0.98 (0.83–1.16)	0.91 (0.76–1.08)	1.09 (0.91–1.31)	0.38	1.03 (1.00–1.07)
**High-fat dairy**						
*n*/N	285/1512	302/1502	288/1532	264/1507		
Median intake	0.4	1.4	2.1	3.6		1.7
Model 1	1 (ref)	1.11 (0.94–1.30)	0.98 (0.83–1.16)	0.91 (0.76–1.09)	0.11	1.00 (0.96–1.04)
Model 2	1 (ref)	1.11 (0.94–1.31)	0.97 (0.82–1.15)	0.94 (0.78–1.13)	0.22	1.00 (0.96–1.04)
**Low-fat dairy**						
*n*/N	273/1517	288/1511	283/1510	295/1515		
Median intake	0.1	1.0	1.8	3.1		1.4
Model 1	1 (ref)	1.07 (0.90–1.26)	1.06 (0.90–1.25)	1.09 (0.92–1.28)	0.31	1.03 (0.99–1.08)
Model 2	1 (ref)	1.08 (0.92–1.28)	1.10 (0.93–1.31)	1.17 (0.99–1.39)	0.06	1.05 (1.01–1.10) *
**Fermented dairy**						
*n*/N	292/1525	284/1501	279/1514	284/1513		
Median intake	1.0	1.8	2.7	4.2		2.2
Model 1	1 (ref)	0.96 (0.82–1.14)	0.93 (0.79–1.10)	0.94 (0.79–1.11)	0.48	1.02 (0.98–1.05)
Model 2	1 (ref)	0.98 (0.83–1.15)	0.95 (0.80–1.13)	1.00 (0.84–1.19)	0.94	1.03 (0.99–1.06)
**High-fat fermented dairy**						
*n*/N	294/1522	279/1498	285/1520	281/1513		
Median intake	0.2	1.1	1.8	3.0		1.3
Model 1	1 (ref)	0.99 (0.84–1.17)	0.95 (0.81–1.12)	0.94 (0.79–1.11)	0.44	1.03 (0.99–1.07)
Model 2	1 (ref)	0.99 (0.83–1.16)	0.94 (0.80–1.11)	0.93 (0.78–1.11)	0.41	1.03 (0.99–1.07)
**Low-fat fermented dairy**						
*n*/N	309/1571	289/1523	258/1463	283/1496		
Median intake	0.0	0.3	0.9	2.1		0.6
Model 1	1 (ref)	0.96 (0.81–1.12)	0.88 (0.75–1.04)	0.93 (0.79–1.10)	0.50	0.99 (0.94–1.05)
Model 2	1 (ref)	0.98 (0.83–1.16)	0.93 (0.79–1.11)	1.01 (0.85–1.19)	0.85	1.01 (0.96–1.07)
**Total milk**						
*n*/N	276/1509	299/1499	271/1437	293/1608		
Median intake	0.1	0.8	1.3	2.4		1.0
Model 1	1 (ref)	1.12 (0.95–1.31)	1.05 (0.89–1.25)	1.03 (0.87–1.22)	0.73	1.00 (0.95–1.06)
Model 2	1 (ref)	1.13 (0.95–1.33)	1.09 (0.92–1.30)	1.09 (0.92–1.29)	0.31	1.02 (0.97–1.08)
**High-fat milk**						
*n*/N	600/3002	148/860	226/1141	165/1050		
Median intake	0.0	0.1	0.2	1.0		0.0
Model 1	1 (ref)	0.90 (0.75–1.08)	1.01 (0.87–1.18)	0.79 (0.66–0.94)	0.02	0.87 (0.78–0.97) *
Model 2	1 (ref)	0.94 (0.78–1.13)	1.03 (0.88–1.21)	0.81 (0.67–0.97)	0.04	0.88 (0.79–0.99) *
**Low-fat milk**						
*n*/N	274/1583	297/1488	294/1490	274/1492		
Median intake	0.0	0.5	1.1	2.2		0.8
Model 1	1 (ref)	1.19 (1.01–1.40)	1.19 (1.00–1.40)	1.09 (0.92–1.29)	0.45	1.05 (0.99–1.11)
Model 2	1 (ref)	1.19 (1.01–1.41)	1.20 (1.02–1.43)	1.14 (0.96–1.36)	0.20	1.07 (1.01–1.13) *
**Total yogurt**						
*n*/N	425/2050	279/1480	221/1231	214/1292		
Median intake	0.0	0.3	0.6	1.0		0.4
Model 1	1 (ref)	0.89 (0.77–1.04)	0.93 (0.78–1.10)	0.80 (0.67–0.94)	0.00	0.88 (0.78–0.98) *
Model 2	1 (ref)	0.92 (0.79–1.08)	1.00 (0.84–1.19)	0.84 (0.71–0.99)	0.05	0.92 (0.82–1.02)
**High-fat yogurt**						
*n*/N	908/4596	69/514	100/500	62/443		
Median intake	0.0	0.1	0.3	0.7		0.0
Model 1	1 (ref)	0.69 (0.54–0.88)	1.04 (0.84–1.28)	0.68 (0.52–0.89)	0.003	0.66 (0.50–0.88) **
Model 2	1 (ref)	0.70 (0.54–0.89)	1.04 (0.84–1.28)	0.70 (0.54–0.91)	0.005	0.67 (0.51–0.89) **
**Low-fat yogurt**						
*n*/N	457/2354	267/1377	205/1070	210/1252		
Median intake	0.0	0.1	0.5	1.0		0.1
Model 1	1 (ref)	1.06 (0.91–1.24)	1.04 (0.87–1.23)	0.94 (0.79–1.11)	0.17	0.94 (0.84–1.06)
Model 2	1 (ref)	1.10 (0.93–1.28)	1.10 (0.92–1.31)	0.99 (0.83–1.17)	0.54	0.99 (0.88–1.11)
**Total cheese**						
*n*/N	272/1501	287/1517	270/1501	310/1534		
Median intake	0.5	1.2	2.0	3.1		1.5
Model 1	1 (ref)	1.04 (0.88–1.23)	0.97 (0.82–1.15)	1.09 (0.92–1.29)	0.46	1.05 (1.01–1.08) *
Model 2	1 (ref)	1.04 (0.88–1.23)	0.98 (0.83–1.17)	1.11 (0.94–1.33)	0.32	1.05 (1.01–1.09) *
**High-fat cheese**						
*n*/N	281/1536	282/1502	286/1503	290/1512		
Median intake	0.1	1.1	1.7	2.9		1.2
Model 1	1 (ref)	1.06 (0.90–1.25)	1.05 (0.89–1.24)	1.04 (0.88–1.24)	0.74	1.04 (1.00–1.07) *
Model 2	1 (ref)	1.05 (0.89–1.24)	1.03 (0.87–1.21)	1.05 (0.88–1.25)	0.75	1.03 (1.00–1.08)
**Low-fat cheese**						
*n*/N	813/4260	111/625	104/560	111/608		
Median intake	0.0	0.2	0.7	1.8		0.0
Model 1	1 (ref)	1.06 (0.86–1.30)	1.13 (0.91–1.41)	1.09 (0.89–1.35)	0.10	1.05 (0.97–1.14)
Model 2	1 (ref)	1.10 (0.90–1.36)	1.16 (0.93–1.45)	1.17 (0.95–1.44)	0.04	1.06 (0.97–1.14)
**Cream**						
*n*/N	735/3604	140/825	128/849	136/775		
Median intake	0.0	0.07	0.18	0.49		0.0
Model 1	1 (ref)	0.89 (0.74–1.09)	0.88 (0.71–1.07)	1.01 (0.83–1.23)	0.41	1.04 (0.93–1.17)
Model 2	1 (ref)	0.90 (0.74–1.10)	0.89 (0.72–1.09)	1.00 (0.82–1.22)	0.52	1.03 (0.92–1.16)
**Ice cream**						
*n*/N	713/3615	137/792	122/765	167/881		
Median intake	0.0	0.07	0.17	0.28		0.0
Model 1	1 (ref)	0.94 (0.78–1.13)	0.87 (0.71–1.07)	0.95 (0.80–1.13)	0.58	0.94 (0.71–1.26)
Model 2	1 (ref)	0.93 (0.77–1.12)	0.86 (0.70–1.05)	0.93 (0.78–1.11)	0.50	0.94 (0.70–1.26)

Continuous analysis in servings/day: milk, 200 mL; yogurt, 150 mL; cheese, 20 g; cream 3 g; ice cream, 50 g. Combined total dairy category: liquid dairy products, 200 mL; cheese, 20 g. Model 1 included age (continuous), sex and energy intake (continuous). Model 2 was additionally adjusted for education (3 categories), smoking (3 categories), physical activity (continuous), alcohol consumption (4 categories), family history of diabetes (yes/no, RS-I and RS-II only) and food groups associated with type 2 diabetes, including intakes of fruit, vegetables, wholegrains, legumes, nuts, tea, coffee, red meat and sugar-sweetened beverages (SSB) (continuous). HR, Hazard Ratio. *p* value significance level: * 0.05, ** 0.01, *** 0.001.

**Table 3:** Associations of dairy product types and longitudinal insulin resistance in the Rotterdam Studies (*n* = 6593).

	Pooled Effect Estimates					
	Intake Range Categories					Continuous
	Q1	Q2	Q3	Q4		
	B	B (95% CI)	B (95% CI)	B (95% CI)	P_trend_	B (95% CI)
**Total dairy**						
*n*	1650	1642	1647	1654		
Median intake	1.6	2.8	3.9	5.6		3.3
Model 1	ref	0.01 (−0.03, 0.05)	−0.02 (−0.05, 0.02)	0.02 (−0.02, 0.06)	0.37	0.00 (−0.01, 0.01)
Model 2	ref	0.02 (−0.02, 0.05)	0.00 (−0.04, 0.04)	0.04 (0.00, 0.08)	0.07	0.00 (0.00, 0.01)
**High-fat dairy**						
*n*	1644	1663	1642	1644		
Median intake	0.4	1.4	2.2	3.6		1.7
Model 1	ref	0.02 (−0.02, 0.06)	−0.03 (−0.07, 0.00)	−0.03 (−0.08, 0.01)	0.03 *	−0.01 (−0.01, 0.00)
Model 2	ref	0.02 (−0.02, 0.06)	−0.03 (−0.07, 0.01)	−0.03 (−0.07, 0.01)	0.06	−0.01 (−0.02, 0.00)
**Low-fat dairy**						
*n*	1652	1655	1655	1631		
Median intake	0.1	1	1.7	3		1.3
Model 1	ref	0.02 (−0.02, 0.06)	0.05 (0.02, 0.09)	0.05 (0.01, 0.09)	0.01 **	0.01 (0.00, 0.02) *
Model 2	ref	0.02 (−0.01, 0.06)	0.06 (0.03, 0.10)	0.06 (0.03, 0.10)	0.0003 ***	0.02 (0.01, 0.03) **
**Fermented dairy**						
*n*	1653	1645	1644	1651		
Median intake	1	1.8	2.7	4.2		2.2
Model 1	ref	−0.03 (−0.07, 0.01)	−0.02 (−0.06, 0.02)	−0.04 (−0.08, 0.00)	0.10	−0.01 (−0.02, 0.00)
Model 2	ref	−0.02 (−0.06, 0.02)	0.00 (−0.04, 0.04)	−0.01 (−0.05, 0.03)	0.77	0.00 (−0.01, 0.00)
**High-fat fermented dairy**						
*n*	1658	1625	1648	1662		
Median intake	0.2	1.1	1.8	3		1.4
Model 1	ref	0.02 (−0.02, 0.06)	−0.02 (−0.06, 0.02)	−0.02 (−0.06, 0.02)	0.22	0.00 (−0.01, 0.01)
Model 2	ref	0.02 (−0.02, 0.06)	−0.01 (−0.05, 0.02)	−0.01 (−0.05, 0.03)	0.49	0.00 (−0.01, 0.01)
**Low-fat fermented dairy**						
*n*	1718	1662	1624	1589		
Median intake	0	0.3	0.9	2		0.5
Model 1	ref	0.04 (0.00, 0.08)	0.03 (−0.01, 0.06)	0.00 (−0.04, 0.04)	0.31	−0.01 (−0.02, 0.00)
Model 2	ref	0.05 (0.01, 0.08)	0.04 (0.00, 0.08)	0.02 (−0.01, 0.06)	0.70	0.00 (−0.01, 0.01)
**Total milk**						
*n*	1633	1627	1752	1581		
Median intake	0.1	0.8	1.3	2.4		1.0
Model 1	ref	0.03 (−0.01, 0.07)	0.03 (−0.01, 0.07)	0.05 (0.01, 0.09)	0.02 *	0.01 (0.00, 0.02)
Model 2	ref	0.03 (−0.01, 0.07)	0.03 (0.00, 0.07)	0.05 (0.01, 0.09)	0.02 *	0.01 (0.00, 0.02)
**High-fat milk**						
*n*	3278	942	1146	1227		
Median intake	0	0.05	0.2	0.9		0.003
Model 1	ref	0.01 (−0.03, 0.05)	−0.02 (−0.05, 0.02)	0.00 (−0.04, 0.04)	0.46	−0.02 (−0.04, 0.00)
Model 2	ref	0.00 (−0.04, 0.04)	−0.03 (−0.06, 0.01)	0.00 (−0.04, 0.03)	0.46	−0.02 (−0.04, 0.00)
**Low-fat milk**						
*n*	1736	1604	1620	1633		
Median intake	0	0.5	1.1	2.2		0.8
Model 1	ref	0.03 (−0.01, 0.06)	0.03 (−0.01, 0.06)	0.07 (0.03, 0.11)	0.002 **	0.02 (0.01, 0.04) ***
Model 2	ref	0.02 (−0.02, 0.06)	0.02 (−0.02, 0.06)	0.07 (0.03, 0.11)	0.001 **	0.02 (0.01, 0.04) ***
**Total yogurt**						
*n*	2259	1606	1402	1326		
Median intake	0	0.3	0.6	1.0		0.4
Model 1	ref	0.04 (0.00, 0.07)	−0.01 (−0.05, 0.03)	−0.03 (−0.07, 0.00)	0.02 *	−0.03 (−0.05, −0.01) **
Model 2	ref	0.04 (0.01, 0.08)	0.00 (−0.04, 0.04)	−0.01 (−0.05, 0.02)	0.18	−0.02 (−0.04, 0.00)
**High-fat yogurt**						
*n*	5021	540	470	562		
Median intake	0	0.1	0.3	0.7		0.0
Model 1	ref	−0.01 (−0.06, 0.03)	0.00 (−0.05, 0.05)	−0.11 (−0.17, −0.06)	0.0001 ***	−0.08 (−0.13, −0.03) **
Model 2	ref	−0.01 (−0.06, 0.04)	0.00 (−0.05, 0.05)	−0.10 (−0.16, −0.05)	0.0003 ***	−0.08 (−0.13, −0.03) **
**Low-fat yogurt**						
*n*	2593	1412	1366	1222		
Median intake	0	0.1	0.5	1.0		0.14
Model 1	ref	0.03 (−0.01, 0.06)	0.05 (0.02, 0.09)	0.00 (−0.04, 0.03)	0.71	−0.02 (−0.04, 0.01)
Model 2	ref	0.03 (0.00, 0.07)	0.07 (0.03, 0.10)	0.01 (−0.02, 0.05)	0.52	0.00 (−0.03, 0.02)
**Total cheese**						
*n*	1650	1633	1670	1640		
Median intake	0.5	1.2	2.0	3.1		1.5
Model 1	ref	0.01 (−0.03, 0.05)	−0.01 (−0.05, 0.02)	−0.01 (−0.05, 0.03)	0.62	0.00 (−0.01, 0.01)
Model 2	ref	0.02 (−0.02, 0.06)	0.00 (−0.04, 0.04)	0.01 (−0.03, 0.05)	0.62	0.00 (−0.01, 0.01)
**High-fat cheese**						
*n*	1691	1607	1652	1643		
Median intake	0.2	1.1	1.7	3.0		1.3
Model 1	ref	0.01 (−0.02, 0.05)	−0.01 (−0.04, 0.03)	−0.01 (−0.05, 0.03)	0.52	0.00 (−0.01, 0.01)
Model 2	ref	0.01 (−0.02, 0.05)	0.00 (−0.04, 0.04)	0.00 (−0.04, 0.04)	0.92	0.00 (−0.01, 0.01)
**Low-fat cheese**						
*n*	4682	664	647	600		
Median intake	0	0.2	0.7	1.8		0.0
Model 1	ref	0.02 (−0.03, 0.07)	0.00 (−0.05, 0.05)	−0.01 (−0.05, 0.04)	0.83	0.00 (−0.02, 0.02)
Model 2	ref	0.02 (−0.02, 0.07)	0.01 (−0.04, 0.06)	0.02 (−0.03, 0.07)	0.50	0.01 (−0.01, 0.03)
**Cream**						
*n*	3977	880	829	907		
Median intake	0	0.07	0.2	0.5		0.0
Model 1	ref	−0.03 (−0.07, 0.02)	−0.06 (−0.10, −0.01)	−0.07 (−0.11, −0.02)	0.89	−0.01 (−0.04, 0.01)
Model 2	ref	−0.03 (−0.07, 0.02)	−0.05 (−0.09, 0.00)	−0.07 (−0.12, −0.03)	0.77	−0.02 (−0.05, 0.01)
**Ice cream**						
*n*	3963	851	956	823		
Median intake	0	0.07	0.2	0.3		0.0
Model 1	ref	0.04 (0.00, 0.08)	0.04 (0.00, 0.08)	0.03 (−0.01, 0.07)	0.05	0.05 (0.00, 0.10) *
Model 2	ref	0.03 (−0.02, 0.07)	0.02 (−0.02, 0.07)	0.01 (−0.03, 0.05)	0.52	0.04 (−0.01, 0.08)

Model 1 included age (continuous), sex and energy intake (continuous). Model 2 was additionally adjusted for education (3 categories), smoking (3 categories), physical activity (continuous), alcohol consumption (4 categories), family history of diabetes (yes/no, RS-I and RS-II only) and intakes of fruit, vegetables, wholegrains, legumes, nuts, tea, coffee, red meat and sugar-sweetened beverages (continuous). Model 4 was additionally adjusted for longitudinal waist circumference. Betas from log-transformed HOMA-IR. *p* value significance level: * 0.05, ** 0.01, *** 0.001.

## Data Availability

Data can be obtained upon request. Requests should be directed towards the management team of the Rotterdam Study (datamanagement.ergo@erasmusmc.nl), which has a protocol for approving data requests. Due to restrictions based on privacy regulations and informed consent of the participants, data cannot be made freely available in a public repository. Analytic code (R syntax) will be made available upon request pending approval by the authors.

## Supplementary Materials

The following supporting information can be downloaded at: https://www.mdpi.com/article/10.3390/nu14030415/s1, Figure S1: Flowchart for inclusion of participants in the analyses for prediabetes and longitudinal insulin resistance; Table S1. Grouping of dairy products included for the present analysis of the Rotterdam Studies; Table S2. Missing values of covariates in participants before imputation; Table S3. Baseline characteristics of study population (*n* = 6770) by specific dairy types; Table S4. Baseline characteristics of study population by sub-cohort (*n* = 6770); Table S5. Baseline characteristics of the study population, stratified by whether or not participants were included in the analysis of this study; Figure S2: Non-linear relationship of dairy product types and longitudinal HOMA-IR by sub-cohort; Table S6. Associations of dairy product types and prediabetes in the Rotterdam Studies, pooled and by sub-cohort (*n* = 6053); Table S7. Associations of dairy product types and longitudinal insulin resistance in the Rotterdam Studies, pooled and by sub-cohort (*n* = 6593); Table S8. Associations of dairy product types and prediabetes risk in the Rotterdam Studies, stratified by sex and baseline WC (*n* = 6053); Table S9. Associations of dairy product types and longitudinal insulin resistance in the Rotterdam Studies, stratified by sex, age and WC (*n* = 6593); Table S10. Sensitivity analyses of associations of dairy product types and prediabetes risk in the Rotterdam Studies (*n* = 6053); Table S11. Sensitivity analyses of associations of dairy product types and longitudinal insulin resistance in the Rotterdam Studies (*n* = 6593); Table S12. Mean dairy intake at baseline and follow-up in a subsample of RS-I and RS-II (*n* = 1887); Table S13. Associations of dairy product intakes and prediabetes risk using repeated measures of dairy intake as time-dependent exposure (*n* = 6053) or adjusting for dairy intake at follow-up (*n* = 1707); Table S14. Associations of dairy product intakes and longitudinal insulin resistance with repeated measures of dairy intake as a fixed effect in RS-I and RS-II (*n* = 4274).
