# Supplementary material for: Dairy Product Consumption in Relation to Incident Prediabetes and Longitudinal Insulin Resistance in the Rotterdam Study
Source: Nutrients. 2022 Jan 18;14(3):415. doi: 10.3390/nu14030415 (PMC8840212; doi:10.3390/nu14030415)
Supplement: Supplementary file 1 [file nutrients-14-00415-s001.zip › nutrients-1544296-supplementary.pdf]

Supplemental tables and figures

Dairy product consumption in relation to incident prediabetes and longitudinal insulin resistance in the Rotterdam Study

Isabel AL Slurink

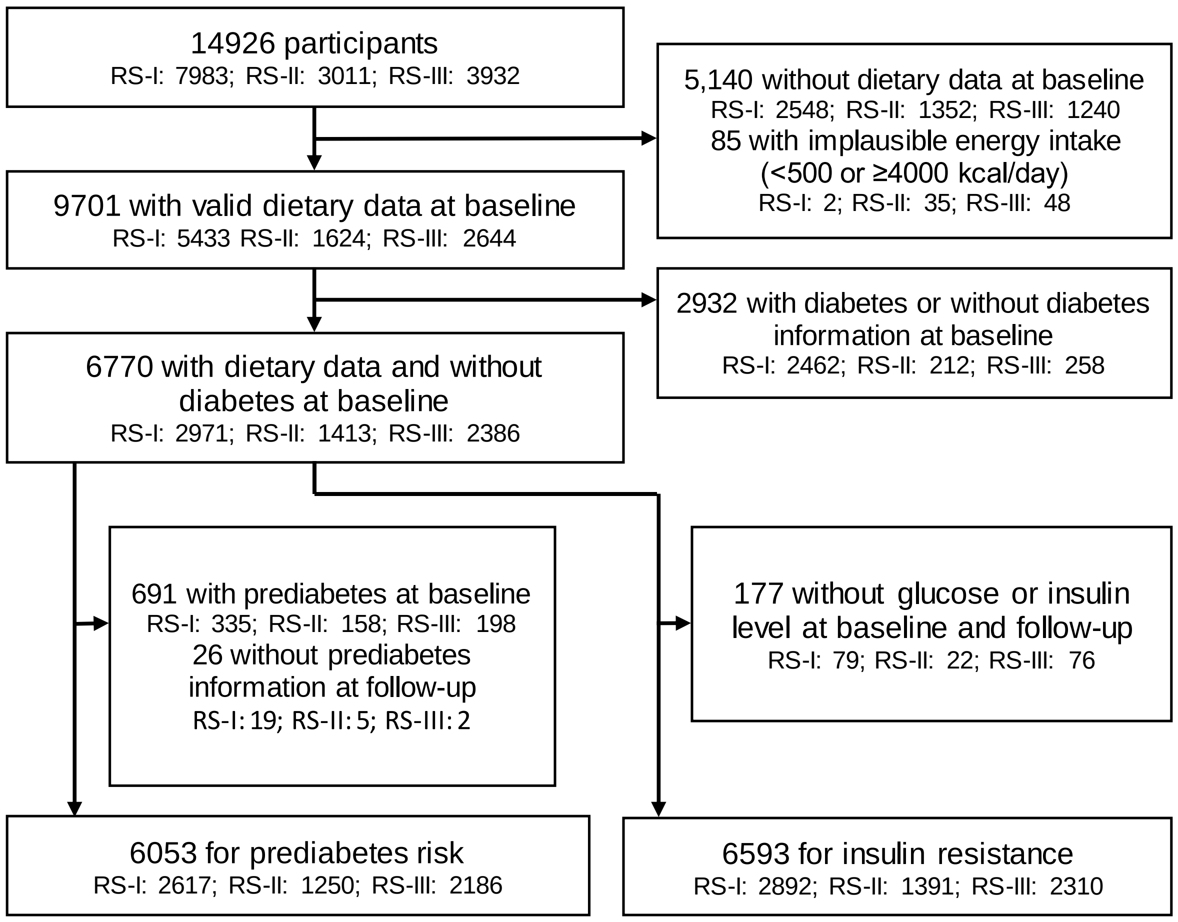

Figure S1

Flow-chart for inclusion of participants in the analyses for prediabetes and longitudinal insulin resistance.

Table S1. *Grouping of dairy products included for the present analysis of the Rotterdam Studies.*

Dairy product	Included dairy types
Total dairy	All dairy products
High-fat[1]	All high-fat dairy products
Low-fat[1]	All low-fat dairy products
Fermented dairy	
High-fat[2]	High-fat yogurt, high-fat cheese, full fat curd, full fat fruit curd
Low-fat	Low-fat yogurt, low-fat cheese, semi-skimmed curd, skimmed curd, semi-skimmed fruit curd, skimmed fruit curd
Milk, all types	
High-fat	Full fat milk, full fat chocolate milk, milk powder, full fat milk added to the coffee
Low-fat	Semi-skimmed milk, skimmed milk, buttermilk, semi-skimmed chocolate milk, skimmed chocolate milk, semi-skimmed milk added to the coffee, skimmed milk added to the coffee
Yogurt	
High-fat	Full fat yogurt, full fat fruit yogurt
Low-fat[3]	Semi-skimmed yogurt, skimmed yogurt, semi-skimmed fruit yogurt skimmed fruit yogurt
Cheese	
High-fat	40+ cheese (e.g., Edam), 48+ cheese (e.g. Gouda, cheddar, cheese spread, goat cheese), full fat luxury cheese (e.g. cream brie, cream cheese, mon chou), cheese cubes, grated cheese, feta, cheese fondue
Low-fat	20+ and 30+ cheese (e.g., cheese spread, cottage cheese), low-fat luxury cheese (e.g. brie, goat cheese)
Cream (high-fat)	Whipped cream, coffee cream, semi-skimmed coffee cream, sour cream, crème fraiche, cooking cream
Ice cream (high-fat)	Ice cream

1 High-fat dairy includes full fat custard for RS-I and RS-III. Low-fat dairy includes skimmed custard for RS-I.

2 Includes mousse and chipolata pudding in RS-III

3 Includes skimmed custard, semi-skimmed curd, skimmed curd, semi-skimmed fruit curd and skimmed fruit curd in RS-III.

Table S2. Missing values of covariates in participants before imputation.

	**Study population** *n*=6770	**Analyses of prediabetes** *n*=6053	**Analyses of HOMA-IR** *n*=5403
Educational level, n (%)	43 (0.6)	34 (0.6)	43 (0.7)
Smoking, n (%)	33 (0.5)	27 (0.4)	33 (0.5)
Physical activity (MET-hours/week), n (%)	265 (3.9)	233 (3.8)	237 (3.6)
Waist circumference, n (%)	312 (4.6)	277 (4.6)	*Repeated measures available*
Cholesterol, n (%)	151 (2.2)	143 (2.4)	*Repeated measures available*
Hypertension, n (%)	58 (0.9)	52 (0.9)	*Repeated measures available*
Triglycerides, n (%)	238 (3.5)	211 (3.5)	*Repeated measures available*

For all covariates of all analyses, only education level, smoking status, physical activity, and baseline waist circumference had missing values before imputation.

Table S3. Baseline characteristics of study population (*n*=6,770) by specific dairy product types.[1]

	**High-fat milk**		**Low-fat milk**		**High-fat yogurt**		**Low-fat cheese**	
	Zero intake n=3358	T3 n=1181	Q1 n=1787	Q4 n=1667	Zero intake n=5150	T3 n=484	Zero intake n=4804	T3 n=665
Total dairy intake (servings/day)	0 ± 0	1.1 ± 0.58	0 ± 0	2.3 ± 0.6	0 ± 0	0.9 ± 0.3	0	2.1 ± 0.5
Range	0.0 - 0.0	0.2 - 14.4	0.0 - 0.1	1.1 - 12.2	0.0 - 0.0	0.4 - 7.4	0.0	0.9 - 15.0
Age at dietary assessment (years)	61.1 ± 3.6	62.2 ± 4.2	62.1 ± 4.0	61.2 ± 3.8	61.7 ± 3.9	62.8 ± 4.0	62.2 ± 4.0	60.6 ± 3.2
Sex female (%)	60.1	55.3	57.3	55.4	57.9	64.7	54.5	69.0
BMI (kg/m2)	26.9 ± 2.3	26.4 ± 2.2	26.3 ± 2.3	26.8 ± 2.2	26.8 ± 2.2	25.7 ± 2.0	26.5 ± 2.2	27.0 ± 2.5
Waist circumference (cm)	91.5 ± 6.7	90.8 ± 6.5	90.7 ± 6.9	92.0 ± 6.9	91.5 ± 6.7	89.6 ± 5.7	91.4 ± 6.7	90.2 ± 6.9
Education level (%)								
Primary education	10.3	14.9	11.9	12.2	11.6	13.2	13.0	8.3
Lower education	40.7	39.5	40.8	37.1	41.7	39.7	41.0	41.8
Intermediate	29.9	26.9	29.2	32.0	28.5	29.1	29.8	28.2
Higher	19.0	18.8	18.2	18.7	18.2	18.0	16.2	21.7
Smoking (%)								
Never	31.6	32.1	30.5	33.7	31.0	38.8	31.6	34.9
Ever	45.5	42.8	42.4	45.3	46.1	41.1	44.8	45.8
Current	22.9	25.2	27.1	21.0	22.9	20.0	23.6	19.3
Physical activity (MET-hours/week)								
Zutphen Physical Activity Questionnaire	42.5 [17.5, 86.0]	46.3 [19.7, 81.1]	42.0 [15.0, 78.0]	43.0 [17.2, 87.7]	42.9 [17.4, 83.5]	46.0 [19.7, 83.5]	40.5 [16.0, 80.8]	50.3 [22.1, 86.9]
LASA Physical Activity Questionnaire	81.1 [56.1, 113.7]	80.1 [53.5, 114.4]	80.0 [53.2, 112.0]	81.0 [56.7, 112.9]	78.2 [53.4, 109.6]	84.7 [62.0, 126.5]	78.5 [53.4, 111.9]	87.6 [62.9, 116.0]
Family history diabetes mellitus	11.6	14.1	12.1	12.7	12.5	15.1	15.1	5.9
Dietary intake								
Diet score	6.7 ± 1.1	6.7 ± 1.0	6.1 ± 1.1	7.2 ± 1.0	6.6 ± 1.1	6.9 ± 1.1	6.4 ± 1.1	7.4 ± 1.0
Energy intake (kcal/day)	2029 ± 323	2287 ± 342	2057 ± 350	2274 ± 336	2087 ± 331	2288 ± 336	2137 ± 337	2057 ± 303
Total fat intake (E%)	34.6 ± 3.7	35.9 ± 3.4	35.8 ± 4.0	34.3 ± 3.5	34.8 ± 3.7	35.0 ± 3.4	35.7 ± 3.6	32.3 ± 3.3
Total saturated fat intake (E%)	12.8 ± 1.6	14.1 ± 1.6	13.5 ± 1.9	13.1 ± 1.5	12.9 ± 1.6	13.7 ± 1.6	13.6 ± 1.6	11.7 ± 1.4
Total protein intake (E%)	17.0 ± 1.7	16.4 ± 1.5	15.9 ± 1.6	17.7 ± 1.6	16.8 ± 1.7	16.1 ± 1.5	16.3 ± 1.6	18.7 ± 1.8
Carbohydrate intake (E%)	43.9 ± 4.3	44.8 ± 3.8	44.0 ± 4.7	45.1 ± 3.8	44.5 ± 4.3	45.8 ± 3.9	44.2 ± 4.3	45.5 ± 3.9
Calcium intake (mg/day)	1061 ± 236	1238 ± 254	883 ± 223	1465 ± 243	1090 ± 251	1271 ± 237	1069 ± 246	1390 ± 246
Sodium intake (mg/day)	2300 ± 455	2455 ± 456	2247 ± 467	2560 ± 447	2322 ± 464	2403 ± 440	2326 ± 465	2601 ± 477
Alcohol intake (g/day)	14.0 ± 9.8	10.6 ± 8.4	13.4 ± 10.1	11.3 ± 8.7	12.9 ± 9.5	11.0 ± 7.7	13.1 ± 9.6	10.3 ± 7.8
Vegetables (g/day)	216.5 ± 69.3	207.9 ± 68.8	204.0 ± 61.8	215.7 ± 72.3	210.2 ± 68.5	205.5 ± 55.8	204.5 ± 66.1	228.0 ± 84.1
Fruit (g/day)	233.7 ± 100.6	210.9 ± 89.0	208.8 ± 101.0	233.7 ± 97.6	226.2 ± 100.2	235.1 ± 87.2	220.6 ± 97.9	266.7 ± 97.6
Wholegrains (g/day)	116.0 ± 43.4	112.9 ± 41.7	104.6 ± 44.6	126.5 ± 43.0	114.9 ± 42.9	119.8 ± 43.2	111.8 ± 43.7	146.6 ± 43.0
Legumes (g/day)	16.4 ± 13.4	16.2 ± 10.5	17.4 ± 16.2	16.2 ± 10.8	16.2 ± 12.6	12.8 ± 8.2	16.4 ± 12.8	17.6 ± 12.7
Nuts (g/day)	8.5 ± 8.3	8.8 ± 7.8	8.4 ± 8.5	8.3 ± 7.2	8.4 ± 8.0	8.8 ± 8.1	8.5 ± 8.1	8.5 ± 7.1
Red meat (g/day)	93.8 ± 36.5	97.0 ± 33.1	91.2 ± 40.5	97.6 ± 31.9	91.6 ± 36.5	94.6 ± 36.9	97.5 ± 38.6	85.3 ± 34.6
Fish (g/day)	20.5 ± 12.9	20.2 ± 12.1	19.5 ± 13.7	19.6 ± 12.1	20.1 ± 12.8	18.4 ± 11.7	19.5 ± 12.6	22.4 ± 13.3
Tea (g/day)	282.4 ± 161.5	291.6 ± 156.1	297.7 ± 168.2	263.6 ± 147.2	285.8 ± 157.6	250.7 ± 122.3	267.8 ± 148.6	314.3 ± 155.7
Coffee (g/day)	483.2 ± 162.2	500.8 ± 160.7	476.7 ± 169.0	475.4 ± 149.0	471.3 ± 152.1	476.3 ± 141.5	477.2 ± 155.9	481.6 ± 142.2
Sugar sweetened beverages (g/day)	87.9 ± 74.4	111.1 ± 79.6	94.7 ± 78.3	95.5 ± 74.5	94.6 ± 75.9	101.5 ± 72.6	97.0 ± 78.0	75.8 ± 60.0

[1] Dairy product types significantly associated with prediabetes risk and longitudinal insulin resistance.
Values are mean ± SD for continuous variables with a normal distribution (pooled), or median (25th percentile (75th percentile) for continuous variables with a skewed distribution; percentages for categorical variables, based on unimputed data.
Abbreviations: RS, Rotterdam Study; SD, standard deviation; E%, percentage of total energy intake; MET, metabolic equivalent of task, T; Tertile, Q; Quartile.

Table S4. Baseline characteristics of study population by sub-cohort (*n*=6770).

	**RS-I** *n=2971*	**RS-II** *n=1413*	**RS-III** *n=2386*	**Pooled** *n=6770*
Total dairy intake (servings/day)	2.1 ± 1.3	1.9 ± 1.4	1.7 ± 1.3	1.9 ± 0.8
Range	0-12.5	0-12.3	0-15.1	0-15.1
Age at dietary assessment (years)	65.5 ± 6.7	63.6 ± 7.2	56.8 ± 6.4	61.7 ± 3.9
Sex, female (%)	59.6	55.1	59.9	58.7
BMI (kg/m2)	26.0 ± 3.4	27.0 ± 4.0	27.2 ± 4.3	26.6 ± 2.2
Waist circumference (cm)	88.8 ± 10.8	93.0 ± 11.8	92.1 ± 12.1	91.1 ± 6.7
Education level (%)				
Primary education	15.8	7.2	9.5	11.8
Lower education	43.8	45.8	34.5	40.9
Intermediate	30.3	28.8	27.3	28.9
Higher	10.1	18.1	28.7	18.3
Smoking (%)				
Never	33.9	29.1	32.1	32.2
Ever	44.7	47.8	43.9	45.0
Current	21.5	23.1	24.0	22.7
Physical activity (MET-hours/week)				
Zutphen Physical Activity Questionnaire	80.7 [55.6, 116.3]	77.3 [53.0, 104.6]	NA	
LASA Physical Activity Questionnaire	NA	NA	42.9 [17.7, 82.5]	
Family history diabetes mellitus	21.9	15.2	NA	
Dietary intake				
Diet score	6.9 ± 1.8	6.1 ± 1.8	7.0 ± 1.9	6.7 ± 1.1
Energy intake (kcal/day)	1984 ± 502	2154 ± 569	2309 ± 714	2113 ± 333
Total fat intake (E%)	35.0 ± 6.0	39.0 ± 7.0	32.0 ± 6.0	35.1 ± 3.6
Total saturated fat intake (E%)	14.0 ± 3.0	15.0 ± 3.0	11.0 ± 3.0	13.2 ± 1.6
Total protein intake (E%)	17.0 ± 3.0	17.0 ± 3.0	16.0 ± 3.0	16.7 ± 1.7
Carbohydrate intake (E%)	45.0 ± 7.0	42.0 ± 8.0	46.0 ± 7.0	44.5 ± 4.2
Calcium intake (mg/day)	1129 ± 397	1098 ± 464	1093 ± 456	1109 ± 251
Sodium intake (mg/day)	2206 ± 657	2536 ± 1005	2439 ± 860	2344 ± 463
Alcohol intake (g/day)	4.5 [0.4, 15.4]	8.7 [0.7, 22.6]	8.2 [1.5, 19.8]	6.6 [0.7, 18.8]
Vegetables (g/day)	209 ± 99	198 ± 110	251 ± 186	211 ± 69
Fruit (g/day)	239 ± 135	179 ± 161	352 ± 321	228 ± 98
Wholegrains (g/day)	71 ± 72	146 ± 72	132 ± 82	116 ± 43
Legumes (g/day)	18 ± 18	15 ± 29	16 ± 22	16.5 ± 12.5
Nuts (g/day)	7.0 ± 12.4	7.4 ± 13.1	12.7 ± 16.4	8.5 ± 7.9
Red meat (g/day)	115 ± 106	108 ± 66	80 ± 48	93 ± 36
Fish (g/day)	15.9 ± 18.3	20.3 ± 22.7	31.3 ± 29.5	20 ± 13
Tea (g/day)	369 ± 260	349 ± 336	190 ± 236	288 ± 155
Coffee (g/day)	494 ± 241	498 ± 285	416 ± 269	471 ± 152
Sugar sweetened beverages (g/day)	67 ± 109	148 ± 177	102 ± 124	94 ± 74

Values are mean ± SD for continuous variables with a normal distribution (pooled), or median (25th percentile (75th percentile) for continuous variables with a skewed distribution; percentages for categorical variables, based on unimputed data.

Abbreviations: RS, Rotterdam Study; SD, standard deviation; E%, percentage of total energy intake; MET, metabolic equivalent of task.

Table S5. Baseline characteristics of the study population, stratified by whether or not participants were included in the analysis of this study.

	Included participants (n = 6770)	Excluded participants (n = 8162)
Age (years)	62.0 ± 7.8	69.2 ± 11.4
Sex, female (%)	58.7	59.4
Body Mass Index (kg/m2)	26.6 ± 3.9	27.1 ± 4.3
Waist circumference (cm)	90.8 ± 11.6	93.4 ± 12.2
Fasting glucose (mmol/L)	5.5 ± 0.6	6.6 ± 2.2
HOMA-IR	2.9 ± 2.4	5.6 ± 13.0
Physical activity (MET-hours/week)		
LASA questionnaire (RS-I and RS-II)	79.7 [54.7, 112.1]	67.2 [40.2, 97.6]
Zutphen Questionnaire (RS-III)	42.9 [17.7, 82.5]	37.3 [16.5, 80.4]
Education level (%)		
Primary	11.8	24.7
Lower	40.9	38.8
Intermediate	28.9	24.8
Higher	18.3	11.6
Smoking (%)		
Never	32.2	34.0
Ever	45.0	40.4
Current	22.7	25.6

Values are mean ± SD for continuous variables with a normal distribution (pooled), or median [IQR] for continuous variables with a skewed distribution; percentages for categorical variables, based on unimputed data. Excluded participants were those without dietary data at baseline (*n*=5,140), implausible energy intake (*n*=85) or with diabetes or without diabetes information at baseline (*n*=2,932).
Abbreviations: MET, metabolic equivalent of task; RS, Rotterdam Study; SD, standard deviation.

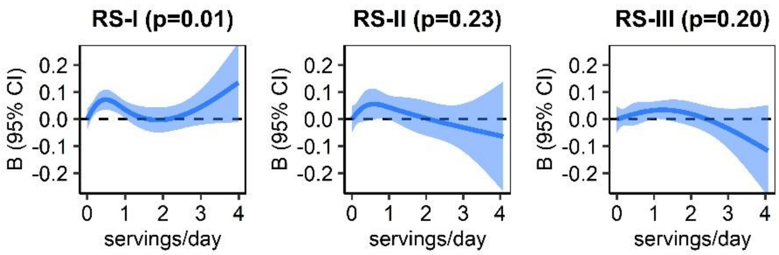

a. Low-fat fermented dairy

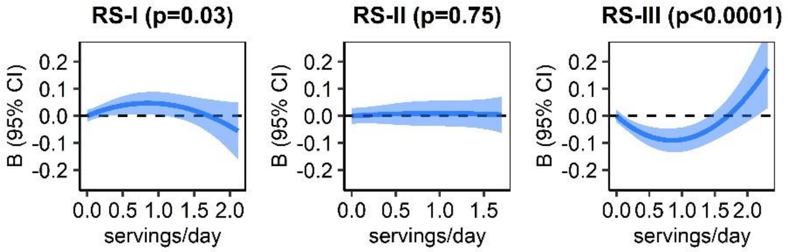

b. High-fat milk

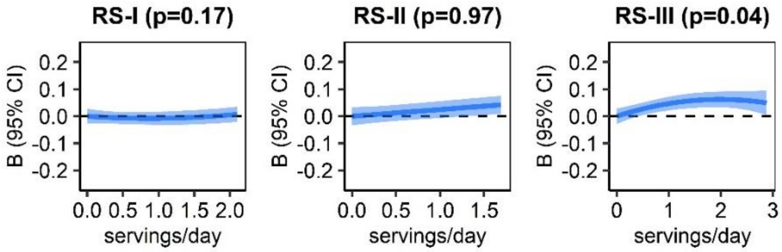

c. Low-fat milk

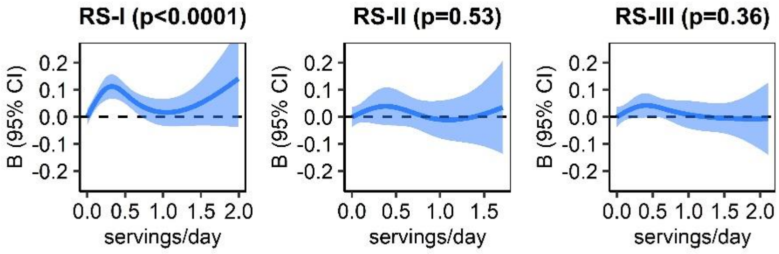

d. Low-fat yogurt

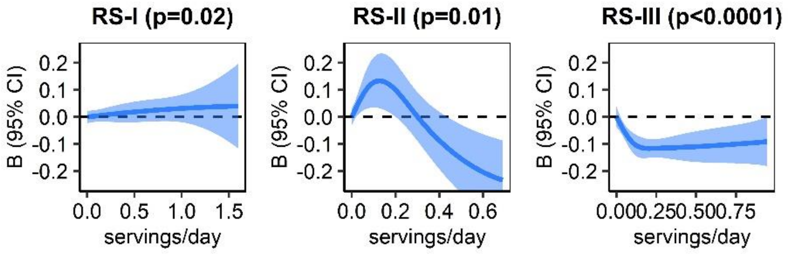

e. Cream

Figure S2

Non-linear relationship of dairy product types and longitudinal HOMA-IR by sub-cohort. Outliers in dairy intakes were excluded (defined as the mean + 3 SD). Dairy product types for which non-linear associations (polynomials or natural splines) were present in at least one sub-cohort are presented.

Table S6. Associations of dairy product types and prediabetes in the Rotterdam Studies, pooled and by sub-cohort (*n*=6053).

	**Pooled effect estimates**	**RS-I**	**RS-II**	**RS-III**
n/N	1139/6053	519/2457	290/1193	330/2139
	Continuous HR (95%CI)	Continuous HR (95%CI)	Continuous HR (95%CI)	Continuous HR (95%CI)
**Total dairy**				
Median intake	3.3	3.5	3.8	2.8
Model 1	1.02 (0.99-1.05)	1.07 (1.02-1.13)**	1.02 (0.97-1.07)	0.92 (0.85-0.98)*
Model 2	1.03 (1.00-1.07)	1.08 (1.03-1.14)**	1.03 (0.98-1.08)	0.95 (0.88-1.02)
**High-fat dairy**				
Median intake	1.7	1.9	2.0	1.1
Model 1	1.00 (0.96-1.04)	1.04 (0.98-1.11)	1.00 (0.95-1.06)	0.91 (0.83-0.99)*
Model 2	1.00 (0.96-1.04)	1.04 (0.98-1.12)	1.00 (0.95-1.06)	0.92 (0.84-1.01)
**Low-fat dairy**				
Median intake	1.4	1.4	1.3	1.4
Model 1	1.03 (0.99-1.08)	1.06 (1.00-1.13)	1.04 (0.96-1.12)	0.96 (0.88-1.06)
Model 2	1.05 (1.01-1.10)*	1.07 (1.01-1.14)*	1.06 (0.99-1.14)	1.00 (0.91-1.10)
**Fermented dairy**				
Median intake	2.2	2.4	2.6	1.9
Model 1	1.02 (0.98-1.05)	1.05 (1.00-1.11)*	1.02 (0.97-1.07)	0.94 (0.86-1.02)
Model 2	1.03 (0.99-1.06)	1.06 (1.00-1.12)*	1.02 (0.97-1.07)	0.97 (0.89-1.06)
**High-fat fermented dairy**				
Median intake	1.3	1.6	1.7	0.9
Model 1	1.03 (0.99-1.07)	1.07 (1.00-1.15)*	1.02 (0.97-1.07)	0.94 (0.85-1.04)
Model 2	1.03 (0.99-1.07)	1.07 (1.00-1.15)*	1.02 (0.96-1.07)	0.95 (0.86-1.06)
**Low-fat fermented dairy**				
Median intake	0.6	0.5	0.5	0.7
Model 1	0.99 (0.94-1.05)	1.01 (0.93-1.10)	0.99 (0.89-1.09)	0.96 (0.86-1.07)
Model 2	1.01 (0.96-1.07)	1.03 (0.94-1.12)	1.01 (0.91-1.11)	1.01 (0.90-1.13)
**Total milk**				
Median intake	1.0	1.2	1.1	0.7
Model 1	1.00 (0.95-1.06)	1.05 (0.98-1.14)	1.00 (0.91-1.11)	0.89 (0.80-1.00)*
Model 2	1.02 (0.97-1.08)	1.06 (0.99-1.15)	1.05 (0.95-1.16)	0.92 (0.82-1.03)
**High-fat milk**				
Median intake	0.0	0.1	0.0	0.0
Model 1	0.87 (0.78-0.97)*	0.95 (0.82-1.10)	0.61 (0.42-0.89)**	0.84 (0.70-1.00)
Model 2	0.88 (0.79-0.99)*	0.95 (0.81-1.10)	0.62 (0.43-0.91)*	0.86 (0.72-1.04)
**Low-fat milk**				
Median intake	0.8	1.0	0.8	0.5
Model 1	1.05 (0.99-1.11)	1.07 (0.99-1.15)	1.06 (0.97-1.17)	0.94 (0.82-1.09)
Model 2	1.07 (1.01-1.13)*	1.08 (1.00-1.16)*	1.11 (1.00-1.23)*	0.96 (0.84-1.11)
**Total yogurt**				
Median intake	0.4	0.3	0.1	0.4
Model 1	0.88 (0.78-0.98)*	0.89 (0.74-1.07)	0.90 (0.72-1.13)	0.85 (0.71-1.01)
Model 2	0.92 (0.82-1.02)	0.89 (0.73-1.07)	0.95 (0.76-1.20)	0.92 (0.77-1.09)
**High-fat yogurt**				
Median intake	0.0	0.0	0.0	0.0
Model 1	0.66 (0.50-0.88)**	0.76 (0.54-1.08)	0.45 (0.19-1.05)	0.51 (0.27-0.97)*
Model 2	0.67 (0.51-0.89)**	0.76 (0.54-1.07)	0.47 (0.20-1.10)	0.55 (0.29-1.04)
**Low-fat yogurt**				
Median intake	0.1	0.1	0.0	0.4
Model 1	0.94 (0.84-1.06)	0.96 (0.77-1.19)	0.98 (0.79-1.22)	0.91 (0.77-1.08)
Model 2	0.99 (0.88-1.11)	0.97 (0.78-1.21)	1.03 (0.82-1.29)	0.98 (0.83-1.16)
**Total cheese**				
Median intake	1.5	1.7	2.0	1.3
Model 1	1.05 (1.01-1.08)**	1.11 (1.04-1.18)**	1.03 (0.98-1.08)	0.98 (0.89-1.08)
Model 2	1.05 (1.01-1.09)**	1.11 (1.04-1.19)**	1.02 (0.97-1.08)	1.00 (0.91-1.10)
**High-fat cheese**				
Median intake	1.2	1.5	1.6	0.8
Model 1	1.04 (1.00-1.07)*	1.08 (1.02-1.16)*	1.03 (0.98-1.08)	0.96 (0.87-1.07)
Model 2	1.03 (1.00-1.08)	1.08 (1.01-1.16)*	1.02 (0.97-1.08)	0.97 (0.87-1.08)
**Low-fat cheese**				
Median intake	0.0	0.0	0.0	0.1
Model 1	1.05 (0.97-1.14)	1.10 (0.97-1.26)	1.00 (0.86-1.16)	1.03 (0.89-1.20)

Model 2	1.06 (0.97-1.14)	1.11 (0.97-1.27)	1.00 (0.88-1.14)	1.07 (0.92-1.24)
**Cream**				
Median intake	0.0	0.0	0.0	0.1
Model 1	1.04 (0.93-1.17)	1.06 (0.94-1.19)	0.93 (0.52-1.66)	0.91 (0.59-1.41)
Model 2	1.03 (0.92-1.16)	1.05 (0.93-1.18)	0.89 (0.50-1.59)	0.92 (0.59-1.42)
**Ice cream**				
Median intake	0.0	0.0	0.0	0.1
Model 1	0.94 (0.71-1.26)	0.83 (0.38-1.81)	0.93 (0.63-1.35)	1.04 (0.61-1.77)
Model 2	0.94 (0.70-1.26)	0.75 (0.34-1.65)	0.96 (0.65-1.43)	1.00 (0.58-1.73)

[1] Continuous analyses in servings/day: milk, 200 mL; yogurt, 150 mL; cheese, 20 g; cream 3 g; ice cream, 50 g. Combined total dairy category: liquid dairy products, 200 mL; cheese, 20g. Model 1 included age (continuous), sex and energy intake (continuous). Model 2 was additionally adjusted for education (3 categories), smoking (3 categories), physical activity (continuous), alcohol consumption (4 categories), family history of diabetes (yes/no, RS-I and RS-II only) and food groups associated with type 2 diabetes including intakes of fruit, vegetables, wholegrains, legumes, nuts, tea, coffee, red meat, and sugar-sweetened beverages (SSB) (continuous). HR, Hazard Ratio. P value significance level: *.05, **.01, ***.001

Table S7. Associations of dairy product types and longitudinal insulin resistance in the Rotterdam Studies, pooled and by sub-cohort (n = 6593).

	**Pooled effect estimates** *n*=6053	**RS-I** *n*=2892	**RS-II** *n*=1391	**RS-III** *n*=2310
	Continuous B (95%CI)	Continuous B (95%CI)	Continuous B (95%CI)	Continuous B (95%CI)
**Total dairy**				
Median intake	3.3	3.5	3.8	2.8
Model 1	0.00 (-0.01,0.01)	0.00 (-0.01,0.01)	0.01 (-0.01,0.02)	0.00 (-0.02,0.01)
Model 2	0.00 (0.00,0.01)	0.00 (-0.01,0.02)	0.01 (-0.01,0.02)	0.00 (-0.01,0.01)
**High-fat dairy**				
Median intake	1.7	1.9	2.0	1.1
Model 1	-0.01 (-0.01,0.00)	-0.01 (-0.02,0.01)	0.00 (-0.01,0.01)	-0.01 (-0.03,0.00)
Model 2	-0.01 (-0.02,0.00)	-0.01 (-0.02,0.01)	0.00 (-0.02,0.01)	-0.01 (-0.03,0.00)
**Low-fat dairy**				
Median intake	1.3	1.3	1.3	1.4
Model 1	0.01 (0.00,0.02)	0.01 (-0.01,0.02)	0.02 (0.00,0.04)	0.01 (-0.01,0.03)
Model 2	0.02 (0.01,0.03)**	0.01 (0.00,0.03)	0.02 (0.00,0.04)*	0.02 (0.00,0.04)*
**Fermented dairy**				
Median intake	2.2	2.4	2.6	1.9
Model 1	-0.01 (-0.02,0.00)	-0.01 (-0.02,0.01)	0.00 (-0.01,0.01)	-0.01 (-0.03,0.00)
Model 2	0.00 (-0.01,0.00)	0.00 (-0.02,0.01)	0.00 (-0.02,0.01)	-0.01 (-0.02,0.01)
**High-fat fermented dairy**				
Median intake	1.4	1.6	1.7	0.8
Model 1	0.00 (-0.01,0.01)	-0.01 (-0.02,0.01)	0.00 (-0.01,0.01)	-0.01 (-0.03,0.01)
Model 2	0.00 (-0.01,0.01)	0.00 (-0.02,0.01)	0.00 (-0.02,0.01)	-0.01 (-0.03,0.01)
**Low-fat fermented dairy**				
Median intake	0.5	0.5	0.5	0.7
Model 1	-0.01 (-0.02,0.00)	-0.01 (-0.03,0.01)	-0.01 (-0.04,0.01)	-0.02 (-0.04,0.01)
Model 2	0.00 (-0.01,0.01)	0.00 (-0.02,0.02)	0.00 (-0.03,0.02)	0.00 (-0.02,0.02)
**Total milk**				
Median intake	1.0	1.2	1.1	0.7
Model 1	0.01 (0.00,0.02)	0.00 (-0.02,0.02)	0.03 (0.01,0.06)*	0.01 (-0.01,0.03)
Model 2	0.01 (0.00,0.02)	0.00 (-0.02,0.02)	0.03 (0.01,0.06)**	0.01 (-0.01,0.02)
**High-fat milk**				
Median intake	0.003	0.06	0.04	0
Model 1	-0.02 (-0.04,0.00)	-0.01 (-0.05,0.02)	-0.02 (-0.07,0.04)	-0.02 (-0.05,0.00)
Model 2	-0.02 (-0.04,0.00)	-0.02 (-0.05,0.02)	-0.02 (-0.08,0.04)	-0.02 (-0.05,0.00)
**Low-fat milk**				
Median intake	0.8	0.9	0.8	0.5
Model 1	0.02 (0.01,0.04)***	0.01 (-0.01,0.03)	0.04 (0.01,0.06)**	0.04 (0.01,0.07)**
Model 2	0.02 (0.01,0.04)***	0.01 (-0.01,0.03)	0.04 (0.01,0.06)**	0.04 (0.01,0.06)**
**Total yogurt**				
Median intake	0.4	0.3	0.07	0.4
Model 1	-0.03 (-0.05,-0.01)**	-0.01 (-0.06,0.03)	-0.02 (-0.07,0.03)	-0.04 (-0.07,-0.01)**
Model 2	-0.02 (-0.04,0.00)	-0.01 (-0.05,0.04)	-0.01 (-0.06,0.05)	-0.03 (-0.06,0.00)
**High-fat yogurt**				

Median intake	0	0	0	0
Model 1	-0.08 (-0.13,-0.03)**	-0.05 (-0.12,0.02)	-0.11 (-0.25,0.03)	-0.11 (-0.19,-0.04)**
Model 2	-0.08 (-0.13,-0.03)**	-0.04 (-0.11,0.03)	-0.11 (-0.25,0.03)	-0.11 (-0.18,-0.03)**
**Low-fat yogurt**				
Median intake	0.14	0.14	0	0.4
Model 1	-0.02 (-0.04,0.01)	0.00 (-0.05,0.05)	-0.01 (-0.06,0.05)	-0.03 (-0.06,0.00)
Model 2	0.00 (-0.03,0.02)	0.01 (-0.04,0.06)	0.01 (-0.05,0.06)	-0.01 (-0.04,0.02)
**Total cheese**				
Median intake	1.5	1.7	2	1.3
Model 1	0.00 (-0.01,0.01)	0.00 (-0.02,0.02)	0.00 (-0.01,0.01)	0.00 (-0.02,0.02)
Model 2	0.00 (-0.01,0.01)	0.00 (-0.02,0.02)	0.00 (-0.02,0.01)	0.01 (-0.01,0.02)
**High-fat cheese**				
Median intake	1.3	1.5	1.7	0.8
Model 1	0.00 (-0.01,0.01)	0.00 (-0.02,0.01)	0.00 (-0.01,0.02)	0.00 (-0.02,0.02)
Model 2	0.00 (-0.01,0.01)	0.00 (-0.02,0.02)	0.00 (-0.01,0.01)	0.00 (-0.02,0.02)
**Low-fat cheese**				
Median intake	0	0	0	0.08
Model 1	0.00 (-0.02,0.02)	0.01 (-0.02,0.05)	-0.03 (-0.07,0.02)	0.00 (-0.03,0.04)
Model 2	0.01 (-0.01,0.03)	0.02 (-0.02,0.06)	-0.01 (-0.06,0.03)	0.02 (-0.01,0.05)
**Cream**				
Median intake	0	0	0	0.0821
Model 1	-0.01 (-0.04,0.01)	0.00 (-0.03,0.03)	-0.10 (-0.23,0.04)	-0.11 (-0.19,-0.02)*
Model 2	-0.02 (-0.05,0.01)	0.00 (-0.03,0.03)	-0.13 (-0.26,0.00)	-0.10 (-0.19,-0.02)*
**Ice cream**				
Median intake	0	0	0	0.0671
Model 1	0.05 (0.00,0.10)*	0.00 (-0.17,0.17)	0.05 (0.00,0.11)*	0.05 (-0.05,0.16)
Model 2	0.04 (-0.01,0.08)	-0.04 (-0.21,0.13)	0.06 (0.00,0.11)*	-0.02 (-0.12,0.08)

[1] Continuous analyses in servings/day: milk, 200 mL; yogurt, 150 mL; cheese, 20 g; cream 3 g; ice cream, 50 g. Combined total dairy category: liquid dairy products, 200 mL; cheese, 20g. Model 1 included age (continuous), sex and energy intake (continuous). Model 2 was additionally adjusted for education (3 categories), smoking (3 categories), physical activity (continuous), alcohol consumption (4 categories), family history of diabetes (yes/no, RS-I and RS-II only) and food groups associated with type 2 diabetes including intakes of fruit, vegetables, wholegrains, legumes, nuts, tea, coffee, red meat, and sugar-sweetened beverages (SSB) (continuous). HR, Hazard Ratio. P value significance level: *.05, **.01, ***.001

Interactions

Table S8. Associations of dairy product types and prediabetes risk in the Rotterdam Studies, stratified by sex and baseline WC (*n*=6053).

	Pooled $P_{interaction}$	Strata	HR (95%CI)	RS-I HR (95%CI)	RS-II HR (95%CI)	RS-III HR (95%CI)
Interaction with sex						
High-fat fermented dairy	0.04	Women	1.06 (1.01-1.11)*	1.06 (0.96-1.18)	1.08 (1.02-1.15) *	0.85 (0.71-1.02)
		Men	1.02 (0.96-1.09)	1.10 (1.00-1.21) *	0.95 (0.86-1.05)	1.02 (0.89-1.17)
High-fat cheese	0.02	Women	1.07 (1.02-1.12)*	1.09 (0.98-1.20)	1.08 (1.02-1.15) *	0.87 (0.72-1.04)
		Men	1.03 (0.97-1.10)	1.11 (1.01-1.22) *	0.95 (0.86-1.05)	1.04 (0.91-1.19)
Interaction with WC						
Low-fat fermented dairy	0.01	WC <90 cm	1.02 (0.93-1.12)	1.00 (0.89-1.14)	1.15 (0.95-1.39)	0.92 (0.73-1.16)
		WC ≥90 cm	1.01 (0.94-1.09)	1.06 (0.94-1.20)	0.93 (0.82-1.07)	1.04 (0.90-1.19)
Low-fat cheese	0.04	WC <90 cm	1.04 (0.90-1.21)	1.09 (0.89-1.34)	1.10 (0.82-1.48)	0.85 (0.60-1.20)
		WC ≥90 cm	1.09 (0.98-1.21)	1.17 (0.96-1.42)	0.95 (0.79-1.14)	1.16 (0.97-1.38)
Ice cream	0.01	WC <90 cm	0.53 (0.27-1.04)	0.37 (0.10-1.41)	0.54 (0.17-1.75)	0.65 (0.22-1.96)
		WC ≥90 cm	1.15 (0.83-1.58)	1.52 (0.54-4.26)	1.09 (0.73-1.63)	1.17 (0.62-2.22)

Only stratified models for which the interaction term was stat sign ($p<0.05$) are presented. Models are adjusted for age (continuous), sex, energy intake (continuous), education (3 categories), smoking (3 categories), physical activity (continuous), alcohol consumption (4 categories), family history of diabetes (yes/no, RS-I and RS-II only) and intake of fruits, vegetables, wholegrains, legumes, nuts, tea, coffee, red meat, and sugar-sweetened beverages (SSB) (continuous). HR, Hazard Ratio. P value significance level: *.05, **.01, ***.001

Table S9. Associations of dairy product types and longitudinal insulin resistance in the Rotterdam Studies, stratified by sex, age and WC (*n*=6593).

	Pooled $P_{interaction}$	Strata	B (95%CI)	RS-I B (95%CI)	RS-II B (95%CI)	RS-III B (95%CI)
Interaction with sex						
Low-fat fermented dairy	0.01	Women	-0.01 (-0.03, 0.00)	-0.01 (-0.03, 0.02)	-0.02 (-0.06, 0.01)	-0.01 (-0.04, 0.01)
		Men	0.00 (-0.02, 0.02)	0.01 (-0.02, 0.05)	0.02 (-0.02, 0.07)	-0.01 (-0.04, 0.01)
Yogurt	0.02	Women	-0.04 (-0.06, -0.01)***	-0.02 (-0.07, 0.03)	-0.06 (-0.12, 0.01)	-0.04 (-0.08, -0.01)*
		Men	-0.02 (-0.05, 0.01)	0.03 (-0.04, 0.11)	0.09 (0.00, 0.18)	-0.04 (-0.08, -0.01)*
Low-fat yogurt	0.01	Women	-0.03 (-0.05, 0.00)	0.01 (-0.05, 0.07)	-0.05 (-0.12, 0.02)	-0.03 (-0.07, 0.00)
		Men	-0.01 (-0.04, 0.02)	0.03 (-0.06, 0.12)	0.13 (0.04, 0.23)*	-0.03 (-0.07, 0.00)
Low-fat cheese	0.05	Women	0.00 (-0.02, 0.03)	0.00 (-0.05, 0.05)	-0.01 (-0.07, 0.04)	0.01 (-0.03, 0.04)
		Men	0.01 (-0.02, 0.04)	0.05 (-0.01, 0.11)	-0.03 (-0.10, 0.05)	0.01 (-0.03, 0.04)
Cream	0.03	Women	0.00 (-0.03, 0.04)	0.01 (-0.03, 0.05)	-0.11 (-0.28, 0.07)	-0.02 (-0.14, 0.09)
		Men	-0.03 (-0.07, 0.01)	-0.03 (-0.07, 0.02)	-0.17 (-0.37, 0.04)	-0.02 (-0.14, 0.09)
Ice cream	0.01	Women	0.07 (0.02, 0.13)**	0.03 (-0.21, 0.28)	0.09 (0.03, 0.16)***	0.00 (-0.13, 0.13)
		Men	-0.03 (-0.10, 0.05)	-0.09 (-0.34, 0.16)	-0.03 (-0.13, 0.06)	0.00 (-0.13, 0.13)
Interaction with age						
High-fat fermented dairy	0.02	Age <61	0.00 (-0.01, 0.01)	0.01 (-0.02, 0.03)	0.01 (-0.01, 0.03)	-0.01 (-0.03, 0.01)
		Age ≥61	-0.01 (-0.02, 0.00)	-0.01 (-0.03, 0.01)	-0.01 (-0.03, 0.01)	0.00 (-0.06, 0.05)
Total cheese	0.01	Age <61	0.01 (0.00, 0.02)	0.02 (-0.01, 0.05)	0.01 (-0.01, 0.03)	0.01 (-0.01, 0.02)
		Age ≥61	-0.01 (-0.02, 0.01)	-0.01 (-0.03, 0.02)	-0.01 (-0.03, 0.01)	0.01 (-0.04, 0.06)
High-fat cheese	0.01	Age <61	0.00 (-0.01, 0.02)	0.01 (-0.02, 0.04)	0.01 (-0.01, 0.03)	0.00 (-0.02, 0.02)
		Age ≥61	-0.01 (-0.02, 0.01)	-0.01 (-0.03, 0.02)	-0.01 (-0.03, 0.01)	0.01 (-0.04, 0.06)
Interaction with baseline WC						
Total dairy	0.01	WC <90 cm	0.00 (-0.01, 0.01)	-0.02 (-0.04, 0.00)	0.01 (-0.01, 0.02)	0.00 (-0.01, 0.02)
		WC ≥90 cm	0.00 (-0.01, 0.01)	0.00 (-0.01, 0.02)	0.00 (-0.02, 0.02)	0.01 (-0.01, 0.02)
Low-fat dairy	0.01	WC <90 cm	0.00 (-0.01, 0.01)	-0.01 (-0.03, 0.01)	0.00 (-0.02, 0.03)	0.01 (-0.01, 0.03)
		WC ≥90 cm	0.01 (0.00, 0.03)	0.02 (0.00, 0.04)	0.01 (-0.01, 0.04)	0.00 (-0.02, 0.03)
Fermented dairy	0.02	WC <90 cm	0.00 (-0.01, 0.01)	-0.02 (-0.04, 0.00)	0.00 (-0.01, 0.02)	0.00 (-0.02, 0.02)
		WC ≥90 cm	0.00 (-0.02, 0.01)	0.00 (-0.02, 0.02)	-0.01 (-0.02, 0.01)	0.00 (-0.02, 0.02)
Low-fat fermented dairy	0.02	WC <90 cm	-0.01 (-0.03, 0.00)	-0.01 (-0.04, 0.02)	-0.02 (-0.05, 0.01)	-0.01 (-0.04, 0.01)
		WC ≥90 cm	0.00 (-0.02, 0.02)	0.01 (-0.02, 0.04)	-0.01 (-0.04, 0.03)	-0.01 (-0.04, 0.02)
Total milk	0.01	WC <90 cm	0.00 (-0.02, 0.02)	-0.01 (-0.04, 0.01)	0.02 (-0.02, 0.05)	0.00 (-0.02, 0.03)
		WC ≥90 cm	0.01 (0.00, 0.03)	0.01 (-0.02, 0.04)	0.03 (0.00, 0.06)	0.00 (-0.02, 0.03)
High-fat milk	0.03	WC <90 cm	-0.02 (-0.04, 0.00)	-0.01 (-0.05, 0.04)	-0.08 (-0.17, 0.01)	-0.02 (-0.05, 0.01)
		WC ≥90 cm	0.00 (-0.03, 0.02)	-0.01 (-0.07, 0.04)	-0.01 (-0.08, 0.06)	0.00 (-0.04, 0.04)
Low-fat yogurt	0.03	WC <90 cm	-0.02 (-0.05, 0.00)	0.00 (-0.07, 0.07)	-0.07 (-0.14, 0.01)	-0.02 (-0.06, 0.01)
		WC ≥90 cm	-0.01 (-0.04, 0.02)	0.00 (-0.07, 0.07)	0.05 (-0.02, 0.12)	-0.03 (-0.08, 0.01)

Only stratified models for which the interaction term was stat sign (p<0.05) are presented. Models are adjusted for age (continuous), sex, energy intake (continuous), education (3 categories), smoking (3 categories), physical activity (continuous), alcohol consumption (4 categories), family history of diabetes (yes/no, RS-I and RS-II only) and intake of fruits, vegetables, wholegrains, legumes, nuts, tea, coffee, red meat and sugar-sweetened beverages (SSB) (continuous). HR, Hazard Ratio. P value significance level: *.05, **.01, ***.001

Sensitivity analyses

Table S10. Sensitivity analyses of associations of dairy product types and prediabetes risk in the Rotterdam Studies (n=6053).

	**Pooled effect estimates** *n*=6053						**RS-I** *n*=2457	**RS-II** *n*=1193	**RS-III** *n*=2139
	Intake range categories					Continuous	Continuous	Continuous	Continuous
	**Q1** HR	**Q2** HR (95%CI	**Q3** HR (95%CI	**Q4** HR (95%CI	P_{trend}	HR (95%CI)	HR (95%CI)	HR (95%CI)	HR (95%CI)
**Total dairy**									
Main analysis model 2	1 (ref)	0.98 (0.83-1.16)	0.91 (0.76-1.08)	1.09 (0.91-1.31)	0.38	1.03 (1.00-1.07)	1.08 (1.03-1.14)**	1.03 (0.98-1.08)	0.95 (0.88-1.02)
1. Adjust. longitudinal WC	1 (ref)	0.97 (0.75-1.27)	0.89 (0.73-1.09)	1.03 (0.85-1.27)	0.78	1.02 (0.97-1.08)	1.06 (0.98-1.15)	1.03 (0.95-1.11)	0.94 (0.83-1.05)
2. Adjust. CVD risk factors	1 (ref)	0.98 (0.83-1.16)	0.90 (0.76-1.08)	1.06 (0.88-1.27)	0.64	1.03 (0.99-1.06)	1.07 (1.02-1.13)*	1.03 (0.98-1.08)	0.95 (0.88-1.02)
3. Adjust. other dairy intake	1 (ref)	0.98 (0.83-1.16)	0.91 (0.76-1.08)	1.09 (0.91-1.31)	0.38	1.03 (1.00-1.07)	1.08 (1.03-1.14)**	1.03 (0.98-1.08)	0.95 (0.88-1.02)
4. Exclusion CHD	1 (ref)	1.02 (0.85-1.22)	0.91 (0.76-1.10)	1.09 (0.90-1.33)	0.45	1.03 (1.00-1.07)	1.07 (1.01-1.13)*	1.04 (0.98-1.09)	0.96 (0.89-1.03)
5. Energy adjusted dairy	1 (ref)	1.17 (0.99-1.37)	1.03 (0.87-1.22)	1.04 (0.87-1.23)	0.95	1.01 (0.96-1.06)	1.05 (0.97-1.13)	1.05 (0.96-1.16)	0.90 (0.82-1.00)
**High-fat dairy**									
Main analysis model 2	1 (ref)	1.11 (0.94-1.31)	0.97 (0.82-1.15)	0.94 (0.78-1.13)	0.22	1.00 (0.96-1.04)	1.04 (0.98-1.12)	1.00 (0.95-1.06)	0.92 (0.84-1.01)
1. Adjust. longitudinal WC	1 (ref)	1.10 (0.84-1.43)	0.97 (0.79-1.18)	0.96 (0.78-1.17)	0.55	1.00 (0.94-1.06)	1.03 (0.93-1.14)	1.00 (0.91-1.09)	0.94 (0.81-1.07)
2. Adjust. CVD risk factors	1 (ref)	1.12 (0.95-1.32)	1.01 (0.85-1.19)	0.95 (0.78-1.14)	0.25	1.00 (0.96-1.04)	1.04 (0.98-1.11)	1.00 (0.95-1.06)	0.93 (0.85-1.02)
3. Adjust. other dairy intake	1 (ref)	1.14 (0.97-1.35)	1.01 (0.85-1.19)	0.99 (0.82-1.20)	0.49	1.02 (0.98-1.06)	1.07 (1.00-1.14)	1.01 (0.96-1.08)	0.92 (0.84-1.01)
4. Exclusion CHD	1 (ref)	1.15 (0.97-1.38)	0.98 (0.82-1.18)	0.98 (0.81-1.19)	0.35	1.00 (0.96-1.05)	1.03 (0.96-1.11)	1.01 (0.96-1.07)	0.95 (0.87-1.04)
5. Energy adjusted dairy	1 (ref)	0.96 (0.81-1.13)	0.97 (0.82-1.16)	0.78 (0.66-0.94)	0.01	0.86 (0.77-0.95)**	0.92 (0.81-1.06)	0.69 (0.52-0.92)*	0.82 (0.68-0.98)*
**Low-fat dairy**									
Main analysis model 2	1 (ref)	1.08 (0.92-1.28)	1.10 (0.93-1.31)	1.17 (0.99-1.39)	0.06	1.05 (1.01-1.10)*	1.07 (1.01-1.14)*	1.06 (0.99-1.14)	1.00 (0.91-1.10)
1. Adjust. longitudinal WC	1 (ref)	1.04 (0.79-1.36)	1.04 (0.85-1.27)	1.08 (0.89-1.31)	0.55	1.03 (0.96-1.11)	1.06 (0.95-1.17)	1.05 (0.92-1.19)	0.96 (0.81-1.12)
2. Adjust. CVD risk factors	1 (ref)	1.07 (0.91-1.27)	1.04 (0.87-1.23)	1.14 (0.96-1.35)	0.15	1.04 (1.00-1.09)*	1.06 (1.00-1.13)	1.06 (0.98-1.13)	0.99 (0.90-1.09)
3. Adjust. other dairy intake	1 (ref)	1.09 (0.92-1.29)	1.11 (0.94-1.32)	1.19 (1.00-1.42)	0.04	1.06 (1.01-1.11)**	1.09 (1.02-1.16)*	1.07 (0.99-1.15)	0.98 (0.89-1.08)
4. Exclusion CHD	1 (ref)	1.07 (0.89-1.28)	1.11 (0.93-1.33)	1.17 (0.98-1.41)	0.06	1.05 (1.00-1.10)*	1.07 (1.00-1.15)	1.06 (0.98-1.14)	0.99 (0.89-1.09)
5. Energy adjusted dairy	1 (ref)	1.18 (1.00-1.39)	1.14 (0.96-1.36)	1.16 (0.98-1.38)	0.09	1.06 (1.01-1.12)*	1.07 (1.00-1.15)	1.11 (1.01-1.21)*	0.97 (0.86-1.09)
**Total fermented dairy**									
Main analysis model 2	1 (ref)	0.98 (0.83-1.15)	0.95 (0.80-1.13)	1.00 (0.84-1.19)	0.94	1.03 (0.99-1.06)	1.06 (1.00-1.12)	1.02 (0.97-1.07)	0.97 (0.89-1.06)
1. Adjust. longitudinal WC	1 (ref)	0.94 (0.72-1.23)	0.94 (0.77-1.15)	0.97 (0.80-1.18)	0.99	1.02 (0.96-1.08)	1.05 (0.95-1.15)	1.02 (0.93-1.10)	0.96 (0.83-1.10)
2. Adjust. CVD risk factors	1 (ref)	0.99 (0.84-1.17)	0.94 (0.79-1.12)	0.98 (0.82-1.17)	0.81	1.02 (0.99-1.06)	1.05 (0.99-1.11)	1.02 (0.96-1.07)	0.97 (0.89-1.06)
3. Adjust. other dairy intake	1 (ref)	0.98 (0.83-1.16)	0.96 (0.81-1.14)	1.02 (0.85-1.22)	0.76	1.03 (1.00-1.07)	1.07 (1.01-1.14)*	1.03 (0.97-1.08)	0.97 (0.89-1.05)
4. Exclusion CHD	1 (ref)	0.99 (0.83-1.18)	0.98 (0.82-1.18)	0.99 (0.82-1.19)	1.00	1.02 (0.99-1.06)	1.05 (0.99-1.12)	1.02 (0.97-1.07)	0.98 (0.89-1.07)
5. Energy adjusted dairy	1 (ref)	0.90 (0.77-1.06)	0.96 (0.82-1.14)	0.85 (0.71-1.01)	0.13	0.97 (0.90-1.05)	0.98 (0.88-1.09)	1.00 (0.87-1.16)	0.89 (0.75-1.06)
**High-fat fermented dairy**									
Main analysis model 2	1 (ref)	0.99 (0.83-1.16)	0.94 (0.80-1.11)	0.93 (0.78-1.11)	0.41	1.03 (0.99-1.07)	1.07 (1.00-1.15)	1.02 (0.96-1.07)	0.95 (0.86-1.06)
1. Adjust. longitudinal WC	1 (ref)	0.98 (0.75-1.29)	0.93 (0.76-1.13)	0.95 (0.78-1.15)	0.72	1.02 (0.96-1.09)	1.05 (0.93-1.17)	1.02 (0.93-1.10)	0.96 (0.81-1.13)
2. Adjust. CVD risk factors	1 (ref)	0.99 (0.84-1.17)	0.97 (0.82-1.15)	0.93 (0.78-1.11)	0.36	1.02 (0.99-1.07)	1.06 (1.00-1.14)*	1.02 (0.96-1.08)	0.96 (0.86-1.06)
3. Adjust. other dairy intake	1 (ref)	1.00 (0.84-1.18)	0.95 (0.81-1.13)	0.95 (0.79-1.14)	0.54	1.03 (0.99-1.08)	1.09 (1.02-1.16)	1.03 (0.97-1.08)	0.94 (0.85-1.05)
4. Exclusion CHD	1 (ref)	1.02 (0.85-1.22)	0.95 (0.79-1.13)	0.93 (0.77-1.12)	0.31	1.03 (0.99-1.07)	1.05 (0.97-1.13)	1.03 (0.97-1.08)	0.98 (0.89-1.09)
5. Energy adjusted dairy	1 (ref)	1.01 (0.85-1.19)	1.01 (0.85-1.19)	0.89 (0.75-1.05)	0.13	0.83 (0.65-1.07)	0.93 (0.65-1.31)	0.87 (0.54-1.40)	0.57 (0.32-1.03)
**Low-fat fermented dairy**									
Main analysis model 2	1 (ref)	0.98 (0.83-1.16)	0.93 (0.79-1.11)	1.01 (0.85-1.19)	0.85	1.01 (0.96-1.07)	1.03 (0.94-1.12)	1.01 (0.91-1.11)	1.01 (0.90-1.13)
1. Adjust. longitudinal WC	1 (ref)	0.98 (0.75-1.28)	0.90 (0.74-1.10)	0.96 (0.79-1.16)	0.81	1.00 (0.91-1.10)	1.02 (0.88-1.18)	1.00 (0.83-1.19)	0.97 (0.79-1.16)
2. Adjust. CVD risk factors	1 (ref)	0.96 (0.82-1.13)	0.92 (0.77-1.09)	0.98 (0.83-1.17)	0.99	1.01 (0.95-1.06)	1.01 (0.93-1.10)	1.00 (0.91-1.10)	1.01 (0.90-1.13)
3. Adjust. other dairy intake	1 (ref)	0.97 (0.82-1.14)	0.92 (0.78-1.10)	1.02 (0.86-1.21)	0.68	1.02 (0.97-1.08)	1.05 (0.96-1.15)	1.02 (0.92-1.12)	0.99 (0.88-1.11)
4. Exclusion CHD	1 (ref)	1.01 (0.85-1.21)	0.96 (0.80-1.15)	1.01 (0.84-1.22)	0.97	1.01 (0.95-1.07)	1.04 (0.95-1.14)	1.00 (0.90-1.10)	0.98 (0.87-1.10)
5. Energy adjusted dairy	1 (ref)	0.96 (0.81-1.13)	1.04 (0.89-1.23)	0.92 (0.77-1.09)	0.41	0.99 (0.91-1.07)	0.99 (0.88-1.11)	1.02 (0.88-1.18)	0.95 (0.80-1.12)
**Total milk**									
Main analysis model 2	1 (ref)	1.13 (0.95-1.33)	1.09 (0.92-1.30)	1.09 (0.92-1.29)	0.31	1.02 (0.97-1.08)	1.06 (0.99-1.15)	1.05 (0.95-1.16)	0.92 (0.82-1.03)
1. Adjust. longitudinal WC	1 (ref)	1.08 (0.83-1.41)	1.08 (0.89-1.32)	1.03 (0.85-1.25)	0.77	1.00 (0.91-1.10)	1.05 (0.92-1.20)	1.01 (0.85-1.19)	0.89 (0.72-1.06)

2. Adjust. CVD risk factors	1 (ref)	1.15 (0.97-1.35)	1.09 (0.92-1.29)	1.09 (0.92-1.29)	0.33	1.02 (0.97-1.08)	1.06 (0.98-1.15)	1.06 (0.95-1.18)	0.91 (0.82-1.02)
3. Adjust. other dairy intake	1 (ref)	1.13 (0.96-1.33)	1.10 (0.92-1.30)	1.10 (0.92-1.30)	0.28	1.03 (0.97-1.09)	1.07 (0.99-1.15)	1.06 (0.95-1.18)	0.92 (0.82-1.03)
4. Exclusion CHD	1 (ref)	1.14 (0.96-1.36)	1.11 (0.93-1.34)	1.11 (0.93-1.34)	0.23	1.02 (0.96-1.08)	1.06 (0.97-1.16)	1.06 (0.95-1.17)	0.92 (0.82-1.04)
5. Energy adjusted dairy	1 (ref)	1.18 (1.00-1.39)	1.04 (0.88-1.24)	1.07 (0.90-1.27)	0.68	1.03 (0.97-1.09)	1.07 (0.99-1.15)	1.05 (0.95-1.17)	0.92 (0.82-1.03)
**High-fat milk**									
Main analysis model 2	1 (ref)	0.94 (0.78-1.13)	1.03 (0.88-1.21)	0.81 (0.67-0.97)	0.04	0.88 (0.79-0.99)*	0.95 (0.81-1.10)	0.62 (0.43-0.91)*	0.86 (0.72-1.04)
1. Adjust. longitudinal WC	1 (ref)	0.94 (0.68-1.30)	1.05 (0.88-1.25)	0.83 (0.69-1.00)	0.28	0.87 (0.71-1.05)	0.95 (0.72-1.20)	0.56 (0.28-1.02)	0.84 (0.60-1.12)
2. Adjust. CVD risk factors	1 (ref)	0.94 (0.78-1.13)	1.05 (0.90-1.23)	0.83 (0.69-1.00)	0.12	0.90 (0.80-1.00)	0.95 (0.82-1.11)	0.61 (0.41-0.89)*	0.89 (0.75-1.07)
3. Adjust. other dairy intake	1 (ref)	0.95 (0.79-1.15)	1.06 (0.90-1.24)	0.86 (0.71-1.03)	0.14	0.91 (0.82-1.02)	1.01 (0.86-1.18)	0.65 (0.44-0.94)*	0.86 (0.72-1.04)
4. Exclusion CHD	1 (ref)	0.92 (0.76-1.13)	1.01 (0.85-1.20)	0.82 (0.68-1.00)	0.09	0.89 (0.79-1.00)	0.95 (0.81-1.12)	0.69 (0.48-1.00)	0.88 (0.73-1.06)
5. Energy adjusted dairy	1 (ref)	0.95 (0.80-1.14)	1.00 (0.84-1.19)	0.84 (0.69-1.01)	0.12	0.89 (0.80-0.99)*	0.95 (0.81-1.10)	0.72 (0.52-1.00)	0.87 (0.72-1.05)
**Low-fat milk**									
Main analysis model 2	1 (ref)	1.19 (1.01-1.41)	1.20 (1.02-1.43)	1.14 (0.96-1.36)	0.20	1.07 (1.01-1.13)*	1.08 (1.00-1.16)*	1.11 (1.00-1.23)*	0.96 (0.84-1.11)
1. Adjust. longitudinal WC	1 (ref)	1.15 (0.88-1.51)	1.16 (0.95-1.41)	1.06 (0.87-1.28)	0.79	1.04 (0.94-1.14)	1.06 (0.93-1.20)	1.06 (0.90-1.25)	0.90 (0.69-1.14)
2. Adjust. CVD risk factors	1 (ref)	1.18 (1.00-1.40)	1.18 (0.99-1.39)	1.12 (0.95-1.34)	0.26	1.07 (1.01-1.13)*	1.08 (1.00-1.16)	1.12 (1.01-1.24)*	0.93 (0.80-1.08)
3. Adjust. other dairy intake	1 (ref)	1.20 (1.02-1.42)	1.21 (1.03-1.44)	1.16 (0.97-1.37)	0.16	1.08 (1.02-1.14)*	1.09 (1.01-1.17)*	1.11 (1.01-1.23)*	0.96 (0.83-1.11)
4. Exclusion CHD	1 (ref)	1.19 (0.99-1.42)	1.17 (0.98-1.41)	1.16 (0.96-1.39)	0.17	1.06 (1.00-1.13)*	1.07 (0.99-1.17)	1.10 (1.00-1.22)	0.96 (0.83-1.11)
5. Energy adjusted dairy	1 (ref)	1.11 (0.94-1.32)	1.13 (0.96-1.34)	1.13 (0.96-1.34)	0.10	1.07 (1.01-1.13)*	1.08 (1.00-1.16)	1.11 (1.01-1.23)*	0.96 (0.83-1.11)
**Total yogurt**									
Main analysis model 2	1 (ref)	0.92 (0.79-1.08)	1.00 (0.84-1.19)	0.84 (0.71-0.99)	0.05	0.92 (0.82-1.02)	0.89 (0.73-1.07)	0.95 (0.76-1.20)	0.92 (0.77-1.09)
1. Adjust. longitudinal WC	1 (ref)	0.90 (0.69-1.16)	1.01 (0.83-1.24)	0.85 (0.71-1.03)	0.35	0.92 (0.76-1.11)	0.88 (0.63-1.20)	0.93 (0.63-1.33)	0.95 (0.69-1.25)
2. Adjust. CVD risk factors	1 (ref)	0.90 (0.77-1.06)	0.99 (0.83-1.18)	0.85 (0.71-1.01)	0.07	0.92 (0.83-1.03)	0.90 (0.74-1.08)	0.95 (0.76-1.20)	0.93 (0.78-1.11)
3. Adjust. other dairy intake	1 (ref)	0.92 (0.79-1.08)	0.99 (0.83-1.18)	0.84 (0.71-1.00)	0.05	0.92 (0.82-1.03)	0.91 (0.75-1.10)	0.96 (0.76-1.21)	0.90 (0.76-1.07)
4. Exclusion CHD	1 (ref)	0.98 (0.83-1.16)	1.02 (0.85-1.23)	0.88 (0.73-1.05)	0.12	0.93 (0.82-1.04)	0.90 (0.73-1.10)	0.99 (0.78-1.25)	0.91 (0.77-1.09)
5. Energy adjusted dairy	1 (ref)	0.90 (0.76-1.06)	0.98 (0.83-1.16)	0.87 (0.73-1.03)	0.10	0.91 (0.82-1.02)	0.88 (0.73-1.07)	0.95 (0.75-1.20)	0.92 (0.78-1.09)
**High-fat yogurt**									
Main analysis model 2	1 (ref)	0.70 (0.54-0.89)	1.04 (0.84-1.28)	0.70 (0.54-0.91)	0.005	0.67 (0.51-0.89)**	0.76 (0.54-1.07)	0.47 (0.20-1.10)	0.55 (0.29-1.04)
1. Adjust. longitudinal WC	1 (ref)	0.67 (0.43-1.05)	1.04 (0.83-1.31)	0.73 (0.56-0.95)	0.12	0.65 (0.40-1.05)	0.74 (0.40-1.24)	0.40 (0.07-1.45)	0.52 (0.15-1.29)
2. Adjust. CVD risk factors	1 (ref)	0.69 (0.54-0.89)	1.02 (0.82-1.26)	0.75 (0.58-0.98)	0.02	0.72 (0.55-0.96)*	0.81 (0.58-1.13)	0.49 (0.20-1.16)	0.58 (0.30-1.10)
3. Adjust. other dairy intake	1 (ref)	0.71 (0.55-0.90)	1.05 (0.85-1.30)	0.72 (0.55-0.94)	0.01	0.70 (0.53-0.93)*	0.81 (0.57-1.14)	0.49 (0.21-1.14)	0.53 (0.28-1.01)
4. Exclusion CHD	1 (ref)	0.75 (0.58-0.97)	1.01 (0.81-1.24)	0.70 (0.51-0.96)	0.03	0.72 (0.54-0.96)*	0.81 (0.57-1.16)	0.51 (0.21-1.22)	0.57 (0.31-1.07)
5. Energy adjusted dairy	1 (ref)	0.72 (0.56-0.93)	1.02 (0.82-1.26)	0.74 (0.58-0.95)	0.010	0.67 (0.50-0.89)**	0.75 (0.53-1.06)	0.47 (0.20-1.10)	0.54 (0.28-1.03)
**Low-fat yogurt**									
Main analysis model 2	1 (ref)	1.10 (0.93-1.28)	1.10 (0.92-1.31)	0.99 (0.83-1.17)	0.54	0.99 (0.88-1.11)	0.97 (0.78-1.21)	1.03 (0.82-1.29)	0.98 (0.83-1.16)
1. Adjust. longitudinal WC	1 (ref)	1.08 (0.83-1.41)	1.09 (0.89-1.32)	0.98 (0.82-1.18)	0.73	0.98 (0.80-1.19)	0.95 (0.64-1.36)	0.99 (0.67-1.40)	0.99 (0.73-1.30)
2. Adjust. CVD risk factors	1 (ref)	1.09 (0.93-1.28)	1.08 (0.91-1.28)	0.98 (0.83-1.17)	0.48	0.98 (0.88-1.10)	0.95 (0.76-1.19)	1.02 (0.82-1.27)	0.98 (0.83-1.16)
3. Adjust. other dairy intake	1 (ref)	1.09 (0.93-1.27)	1.09 (0.92-1.30)	0.98 (0.83-1.17)	0.49	0.98 (0.88-1.10)	0.98 (0.79-1.22)	1.04 (0.83-1.30)	0.96 (0.81-1.13)
4. Exclusion CHD	1 (ref)	1.13 (0.96-1.34)	1.11 (0.92-1.33)	1.02 (0.85-1.22)	0.68	0.99 (0.88-1.12)	0.96 (0.75-1.22)	1.06 (0.84-1.34)	0.97 (0.82-1.15)
5. Energy adjusted dairy	1 (ref)	1.05 (0.90-1.24)	1.07 (0.91-1.26)	0.99 (0.83-1.17)	0.69	0.99 (0.88-1.11)	0.97 (0.78-1.20)	1.03 (0.82-1.28)	0.98 (0.83-1.16)
**Total cheese**									
Main analysis model 2	1 (ref)	1.04 (0.88-1.23)	0.98 (0.83-1.17)	1.11 (0.94-1.33)	0.32	1.05 (1.01-1.09)**	1.11 (1.04-1.19)**	1.02 (0.97-1.08)	1.00 (0.91-1.10)
1. Adjust. longitudinal WC	1 (ref)	0.99 (0.75-1.29)	0.93 (0.76-1.14)	1.06 (0.87-1.29)	0.69	1.03 (0.97-1.10)	1.08 (0.97-1.20)	1.02 (0.93-1.11)	0.98 (0.83-1.14)
2. Adjust. CVD risk factors	1 (ref)	1.05 (0.89-1.25)	1.00 (0.84-1.19)	1.09 (0.91-1.30)	0.52	1.04 (1.00-1.08)	1.09 (1.02-1.17)*	1.02 (0.96-1.07)	1.00 (0.91-1.10)
3. Adjust. other dairy intake	1 (ref)	1.04 (0.88-1.23)	0.98 (0.83-1.17)	1.11 (0.94-1.33)	0.31	1.05 (1.01-1.09)*	1.12 (1.04-1.19)**	1.03 (0.97-1.08)	0.99 (0.90-1.09)
4. Exclusion CHD	1 (ref)	1.04 (0.87-1.24)	1.00 (0.83-1.20)	1.08 (0.90-1.30)	0.51	1.04 (1.00-1.08)*	1.09 (1.02-1.18)*	1.02 (0.97-1.08)	1.01 (0.92-1.11)
5. Energy adjusted dairy	1 (ref)	1.00 (0.84-1.19)	1.02 (0.86-1.21)	1.11 (0.93-1.31)	0.25	1.05 (1.01-1.09)*	1.11 (1.04-1.19)***	1.02 (0.97-1.08)	1.00 (0.91-1.10)
**High-fat cheese**									
Main analysis model 2	1 (ref)	1.05 (0.89-1.24)	1.03 (0.87-1.21)	1.05 (0.88-1.25)	0.75	1.03 (1.00-1.08)	1.08 (1.01-1.16)*	1.02 (0.97-1.08)	0.97 (0.87-1.08)
1. Adjust. longitudinal WC	1 (ref)	1.04 (0.79-1.36)	1.00 (0.82-1.22)	1.05 (0.86-1.27)	0.83	1.01 (0.96-1.06)	1.06 (0.94-1.17)*	1.02 (0.93-1.10)	0.97 (0.93-1.14)
2. Adjust. CVD risk factors	1 (ref)	1.05 (0.89-1.25)	1.04 (0.88-1.24)	1.03 (0.86-1.23)	0.89	1.03 (0.99-1.07)	1.07 (1.00-1.15)*	1.02 (0.97-1.08)	0.97 (0.87-1.08)
3. Adjust. other dairy intake	1 (ref)	1.06 (0.90-1.26)	1.04 (0.88-1.23)	1.06 (0.89-1.27)	0.63	1.04 (1.00-1.08)*	1.10 (1.03-1.17)*	1.03 (0.97-1.09)	0.96 (0.86-1.07)
4. Exclusion CHD	1 (ref)	1.07 (0.89-1.28)	1.03 (0.86-1.23)	1.03 (0.85-1.24)	0.99	1.03 (0.99-1.08)	1.06 (0.98-1.14)	1.03 (0.97-1.09)	1.00 (0.90-1.11)
5. Energy adjusted dairy	1 (ref)	1.05 (0.88-1.24)	1.00 (0.85-1.19)	1.06 (0.90-1.26)	0.51	1.04 (1.00-1.08)	1.08 (1.01-1.16)*	1.02 (0.97-1.08)	0.98 (0.88-1.09)

**Low-fat cheese**									
Main analysis model 2	1 (ref)	1.10 (0.90-1.36)	1.16 (0.93-1.45)	1.17 (0.95-1.44)	0.04	1.06 (0.97-1.14)	1.11 (0.97-1.27)	1.00 (0.88-1.14)	1.07 (0.92-1.24)
1. Adjust. longitudinal WC	1 (ref)	1.05 (0.73-1.50)	1.03 (0.81-1.32)	1.07 (0.86-1.32)	0.44	1.02 (0.88-1.19)	1.08 (0.85-1.34)	0.99 (0.73-1.32)	0.98 (0.74-1.24)
2. Adjust. CVD risk factors	1 (ref)	1.08 (0.88-1.34)	1.11 (0.89-1.38)	1.12 (0.91-1.39)	0.12	1.03 (0.95-1.12)	1.07 (0.93-1.23)	0.98 (0.86-1.12)	1.06 (0.91-1.24)
3. Adjust. other dairy intake	1 (ref)	**1.13 (0.91-1.39)**	**1.18 (0.94-1.47)**	**1.19 (0.96-1.47)**	**0.02**	1.07 (0.99-1.16)	1.15 (1.00-1.33)	1.01 (0.89-1.16)	1.05 (0.90-1.22)
4. Exclusion CHD	1 (ref)	1.09 (0.88-1.36)	1.14 (0.90-1.44)	1.14 (0.91-1.42)	0.09	1.05 (0.96-1.14)	1.14 (0.98-1.33)	0.99 (0.86-1.13)	1.03 (0.88-1.22)
5. Energy adjusted dairy	1 (ref)	1.06 (0.86-1.32)	1.23 (1.00-1.52)	1.13 (0.91-1.40)	0.05	1.06 (0.98-1.14)	1.11 (0.97-1.27)	1.00 (0.88-1.14)	1.07 (0.92-1.24)
**Cream**									
Main analysis model 2	1 (ref)	0.90 (0.74-1.10)	0.89 (0.72-1.09)	1.00 (0.82-1.22)	0.52	1.03 (0.92-1.16)	1.05 (0.93-1.18)	0.89 (0.50-1.59)	0.92 (0.59-1.42)
1. Adjust. longitudinal WC	1 (ref)	0.90 (0.66-1.24)	0.94 (0.75-1.17)	1.03 (0.85-1.26)	0.65	1.02 (0.84-1.25)	1.04 (0.84-1.26)	0.88 (0.34-1.90)	0.91 (0.41-1.80)
2. Adjust. CVD risk factors	1 (ref)	0.91 (0.75-1.10)	0.90 (0.73-1.10)	1.03 (0.84-1.26)	0.45	1.04 (0.93-1.16)	1.05 (0.93-1.18)	0.92 (0.51-1.65)	0.97 (0.63-1.49)
3. Adjust. other dairy intake	1 (ref)	0.90 (0.74-1.10)	0.91 (0.74-1.12)	1.04 (0.85-1.28)	0.17	1.09 (0.97-1.22)	1.11 (0.98-1.26)	0.93 (0.52-1.67)	0.86 (0.55-1.35)
4. Exclusion CHD	1 (ref)	0.93 (0.76-1.14)	0.90 (0.73-1.11)	0.96 (0.78-1.19)	0.58	1.02 (0.91-1.16)	1.05 (0.92-1.19)	0.69 (0.33-1.42)	0.90 (0.57-1.42)
5. Energy adjusted dairy	1 (ref)	0.88 (0.72-1.08)	0.94 (0.77-1.15)	0.96 (0.79-1.18)	0.58	1.03 (0.92-1.16)	1.05 (0.93-1.18)	0.90 (0.50-1.60)	0.90 (0.58-1.39)
**Ice cream**									
Main analysis model 2	1 (ref)	0.93 (0.77-1.12)	0.86 (0.70-1.05)	0.93 (0.78-1.11)	0.50	0.94 (0.70-1.26)	0.75 (0.34-1.65)	0.96 (0.65-1.43)	1.00 (0.58-1.73)
1. Adjust. longitudinal WC	1 (ref)	0.87 (0.62-1.20)	0.84 (0.66-1.05)	0.90 (0.76-1.08)	0.57	0.85 (0.51-1.42)	0.60 (0.13-2.22)	0.85 (0.40-1.52)	0.98 (0.36-2.32)
2. Adjust. CVD risk factors	1 (ref)	0.92 (0.76-1.11)	0.88 (0.71-1.08)	0.95 (0.80-1.13)	0.68	0.98 (0.74-1.31)	0.78 (0.35-1.73)	1.01 (0.69-1.49)	1.02 (0.60-1.73)
3. Adjust. other dairy intake	1 (ref)	0.93 (0.77-1.12)	0.87 (0.71-1.06)	0.95 (0.80-1.13)	0.61	0.97 (0.72-1.31)	0.89 (0.40-1.98)	1.01 (0.67-1.50)	0.94 (0.54-1.63)
4. Exclusion CHD	1 (ref)	0.95 (0.78-1.15)	0.89 (0.72-1.11)	0.97 (0.81-1.17)	0.76	0.91 (0.66-1.25)	0.86 (0.37-2.02)	0.86 (0.54-1.35)	1.01 (0.59-1.73)
5. Energy adjusted dairy	1 (ref)	0.91 (0.76-1.09)	0.85 (0.70-1.04)	0.93 (0.78-1.13)	0.37	0.93 (0.70-1.25)	0.73 (0.34-1.56)	0.95 (0.63-1.41)	1.03 (0.60-1.77)

Model 2 included age (continuous), sex and energy intake (continuous), education (3 categories), smoking (3 categories), physical activity (continuous), alcohol consumption (4 categories), family history of diabetes (yes/no, RS-I and RS-II only) and intakes of fruit, vegetables, wholegrains, legumes, nuts, tea, coffee, red meat and sugar-sweetened beverages (continuous). CVD, Cardiovascular Disease; CHD, Coronary Heart Disease; HR, Hazard Ratio; WC, waist circumference. P value significance level: *.05, **.01, ***.001

Table S11. Sensitivity analyses of associations of dairy product types and longitudinal insulin resistance in the Rotterdam Studies (*n*=6593).

	**Pooled effect estimates** *n*=6593						**RS-I** *n*=2892	**RS-II** *n*=1391	**RS-III** *n*=2310
	Intake range categories					Continuous	Continuous	Continuous	Continuous
	Q1 B	Q2 B (95%CI	Q3 B (95%CI	Q4 B (95%CI	P_{trend}	B (95%CI)	B (95%CI)	B (95%CI)	B (95%CI)
**Total dairy**									
Main analysis model 2	ref	0.02 (-0.02,0.05)	0.00 (-0.04,0.04)	0.04 (0.00,0.08)	0.07	0.00 (0.00,0.01)	0.00 (-0.01,0.02)	0.01 (-0.01,0.02)	0.00 (-0.01,0.01)
1. Adjust. longitudinal WC	ref	0.01 (-0.03,0.04)	-0.01 (-0.05,0.02)	0.01 (-0.03,0.04)	0.81	0.00 (-0.01,0.00)	-0.01 (-0.02,0.00)	0.00 (-0.01,0.01)	0.00 (-0.01,0.01)
2. Adjust. CVD risk factors	ref	0.02 (-0.02,0.05)	0.00 (-0.03,0.04)	0.03 (-0.01,0.06)	0.23	0.00 (-0.01,0.01)	0.00 (-0.01,0.01)	0.00 (-0.01,0.01)	0.00 (-0.01,0.01)
3. Adjust. other dairy intake	ref	0.02 (-0.02,0.05)	0.00 (-0.04,0.04)	0.04 (0.00,0.08)	0.07	0.00 (0.00,0.01)	0.00 (-0.01,0.02)	0.01 (-0.01,0.02)	0.00 (-0.01,0.01)
4. Exclusion CHD	ref	0.02 (-0.02,0.06)	0.00 (-0.04,0.04)	0.04 (0.00,0.09)	0.05	0.00 (0.00,0.01)	0.00 (-0.01,0.02)	0.01 (0.00,0.02)	0.00 (-0.01,0.01)
5. Energy adjusted dairy	ref	0.00 (-0.04,0.04)	0.02 (-0.02,0.06)	0.03 (-0.01,0.07)	0.07	0.01 (-0.01,0.02)	0.00 (-0.01,0.02)	0.02 (0.00,0.05)*	0.00 (-0.02,0.02)
**High-fat dairy**									
Main analysis model 2	ref	0.02 (-0.02,0.06)	-0.03 (-0.07,0.01)	-0.03 (-0.07,0.01)	0.06	-0.01 (-0.02,0.00)	-0.01 (-0.02,0.01)	0.00 (-0.02,0.01)	-0.01 (-0.03,0.00)
1. Adjust. longitudinal WC	ref	0.02 (-0.01,0.05)	-0.02 (-0.05,0.02)	-0.01 (-0.05,0.02)	0.18	0.00 (-0.01,0.00)	-0.01 (-0.03,0.00)	0.00 (-0.01,0.01)	0.00 (-0.01,0.01)
2. Adjust. CVD risk factors	ref	0.03 (0.00,0.06)	0.00 (-0.03,0.04)	-0.01 (-0.05,0.03)	0.28	-0.01 (-0.01,0.00)	0.00 (-0.02,0.01)	0.00 (-0.02,0.01)	-0.01 (-0.02,0.01)
3. Adjust. other dairy intake	ref	0.03 (-0.01,0.06)	-0.02 (-0.06,0.02)	-0.02 (-0.06,0.03)	0.23	0.00 (-0.01,0.00)	0.00 (-0.02,0.01)	0.00 (-0.01,0.01)	-0.01 (-0.03,0.01)
4. Exclusion CHD	ref	0.02 (-0.02,0.06)	-0.04 (-0.08,0.00)	-0.03 (-0.07,0.01)	0.075	-0.01 (-0.02,0.00)	-0.01 (-0.03,0.01)	0.00 (-0.01,0.01)	-0.01 (-0.03,0.00)
5. Energy adjusted dairy	ref	-0.02 (-0.06,0.02)	-0.02 (-0.06,0.02)	-0.04 (-0.08,-0.01)	0.02*	-0.03 (-0.05,-0.01)**	-0.02 (-0.05,0.01)	-0.03 (-0.08,0.02)	-0.03 (-0.05,0.00)
**Low-fat dairy**									
Main analysis model 2	ref	0.02 (-0.01,0.06)	0.06 (0.03,0.10)	0.06 (0.03,0.10)	0.0003***	0.02 (0.01,0.03)**	0.00 (-0.01,0.01)	0.02 (0.00,0.04)*	0.02 (0.00,0.04)*
1. Adjust. longitudinal WC	ref	0.00 (-0.03,0.03)	0.02 (-0.01,0.05)	0.01 (-0.02,0.05)	0.31	0.00 (-0.01,0.01)	0.00 (-0.01,0.02)	0.00 (-0.01,0.02)	0.00 (-0.02,0.01)
2. Adjust. CVD risk factors	ref	0.02 (-0.01,0.06)	0.05 (0.01,0.08)	0.04 (0.01,0.08)	0.005**	0.01 (0.00,0.02)	0.00 (-0.01,0.02)	0.02 (0.00,0.04)	0.01 (-0.01,0.02)
3. Adjust. other dairy intake	ref	0.02 (-0.02,0.06)	0.06 (0.02,0.10)	0.06 (0.02,0.10)	0.001***	0.01 (0.00,0.02)**	0.01 (-0.01,0.03)	0.02 (0.00,0.04)*	0.02 (0.00,0.03)
4. Exclusion CHD	ref	0.02 (-0.02,0.05)	0.06 (0.02,0.10)	0.07 (0.03,0.11)	0.0001***	0.02 (0.01,0.03)**	0.01 (0.00,0.03)	0.03 (0.00,0.05)*	0.02 (0.00,0.03)
5. Energy adjusted dairy	ref	0.01 (-0.02,0.05)	0.06 (0.02,0.09)	0.06 (0.02,0.10)	0.0007***	0.02 (0.01,0.03)**	0.01 (-0.01,0.03)	0.03 (0.01,0.05)**	0.02 (0.00,0.04)
**Total fermented dairy**									
Main analysis model 2	ref	-0.02 (-0.06,0.02)	0.00 (-0.04,0.04)	-0.01 (-0.05,0.03)	0.77	0.00 (-0.01,0.00)	-0.01 (-0.02,0.01)	0.00 (-0.02,0.01)	-0.01 (-0.02,0.01)
1. Adjust. longitudinal WC	ref	-0.02 (-0.06,0.01)	-0.01 (-0.05,0.02)	-0.04 (-0.07,0.00)	0.13	-0.01 (-0.01,0.00)	-0.01 (-0.03,0.00)	0.00 (-0.01,0.01)	-0.01 (-0.02,0.01)
2. Adjust. CVD risk factors	ref	-0.01 (-0.05,0.02)	0.00 (-0.03,0.04)	-0.02 (-0.05,0.02)	0.51	0.00 (-0.01,0.00)	0.00 (-0.02,0.01)	-0.01 (-0.02,0.01)	0.00 (-0.02,0.01)
3. Adjust. other dairy intake	ref	-0.02 (-0.05,0.02)	0.00 (-0.04,0.04)	-0.01 (-0.05,0.03)	0.87	0.00 (-0.01,0.01)	0.00 (-0.02,0.01)	0.00 (-0.01,0.01)	0.00 (-0.02,0.01)
4. Exclusion CHD	ref	-0.02 (-0.06,0.02)	-0.01 (-0.05,0.03)	-0.02 (-0.07,0.02)	0.49	0.00 (-0.01,0.01)	0.00 (-0.02,0.01)	0.00 (-0.01,0.01)	-0.01 (-0.02,0.01)
5. Energy adjusted dairy	ref	0.00 (-0.04,0.04)	0.01 (-0.03,0.04)	-0.03 (-0.07,0.01)	0.15	-0.02 (-0.03,0.00)*	-0.01 (-0.04,0.01)	-0.01 (-0.04,0.03)	-0.03 (-0.06,0.00)*
**High-fat fermented dairy**									
Main analysis model 2	ref	0.02 (-0.02,0.06)	-0.01 (-0.05,0.02)	-0.01 (-0.05,0.03)	0.49	0.00 (-0.01,0.01)	0.01 (-0.01,0.02)	0.00 (-0.02,0.01)	-0.01 (-0.03,0.01)
1. Adjust. longitudinal WC	ref	0.03 (0.00,0.06)	0.00 (-0.03,0.03)	0.00 (-0.03,0.04)	0.77	0.00 (-0.01,0.01)	-0.02 (-0.03,0.00)	0.00 (-0.01,0.01)	0.01 (-0.01,0.02)
2. Adjust. CVD risk factors	ref	0.03 (0.00,0.06)	0.01 (-0.02,0.05)	0.00 (-0.04,0.03)	0.72	0.00 (-0.01,0.01)	0.00 (-0.02,0.02)	-0.01 (-0.02,0.01)	0.00 (-0.02,0.02)
3. Adjust. other dairy intake	ref	0.03 (-0.01,0.06)	-0.01 (-0.05,0.03)	0.00 (-0.04,0.04)	0.81	0.00 (-0.01,0.01)	0.00 (-0.02,0.02)	0.00 (-0.01,0.01)	-0.01 (-0.03,0.01)
4. Exclusion CHD	ref	0.02 (-0.02,0.06)	-0.02 (-0.06,0.02)	-0.02 (-0.06,0.03)	0.41	0.00 (-0.01,0.01)	-0.01 (-0.03,0.01)	0.00 (-0.01,0.01)	-0.01 (-0.03,0.01)
5. Energy adjusted dairy	ref	-0.02 (-0.05,0.02)	-0.01 (-0.05,0.03)	-0.05 (-0.09,-0.01)	0.02*	-0.08 (-0.13,-0.03)**	-0.05 (-0.14,0.03)	-0.06 (-0.17,0.04)	-0.12 (-0.21,-0.04)**
**Low-fat fermented dairy**									
Main analysis model 2	ref	0.05 (0.01,0.08)	0.04 (0.00,0.08)	0.02 (-0.01,0.06)	0.70	0.00 (-0.01,0.01)	-0.01 (-0.02,0.01)	0.00 (-0.03,0.02)	0.00 (-0.02,0.02)
1. Adjust. longitudinal WC	ref	0.03 (0.00,0.06)	0.00 (-0.03,0.04)	-0.02 (-0.05,0.02)	0.05	-0.01 (-0.02,0.00)*	-0.01 (-0.02,0.01)	-0.01 (-0.04,0.01)	-0.02 (-0.04,0.00)*
2. Adjust. CVD risk factors	ref	0.03 (0.00,0.06)	0.03 (-0.01,0.06)	0.01 (-0.03,0.05)	0.93	-0.01 (-0.02,0.01)	-0.01 (-0.02,0.01)	-0.01 (-0.03,0.02)	-0.01 (-0.02,0.01)
3. Adjust. other dairy intake	ref	0.05 (0.01,0.08)	0.04 (0.00,0.08)	0.03 (-0.01,0.07)	0.58	0.00 (-0.01,0.01)	0.00 (-0.02,0.02)	0.00 (-0.03,0.02)	0.00 (-0.02,0.02)
4. Exclusion CHD	ref	0.05 (0.01,0.09)	0.04 (0.00,0.08)	0.03 (-0.02,0.07)	0.75	0.00 (-0.01,0.01)	0.00 (-0.02,0.03)	0.00 (-0.03,0.03)	0.00 (-0.02,0.02)
5. Energy adjusted dairy	ref	0.06 (0.02,0.10)	0.05 (0.01,0.09)	0.02 (-0.02,0.05)	0.76	-0.01 (-0.03,0.01)	-0.01 (-0.03,0.02)	0.00 (-0.04,0.03)	-0.02 (-0.05,0.01)
**Total milk**									
Main analysis model 2	ref	0.03 (-0.01,0.07)	0.03 (0.00,0.07)	0.05 (0.01,0.09)	0.02*	0.01 (0.00,0.02)	-0.01 (-0.02,0.01)	0.03 (0.01,0.06)**	0.01 (-0.01,0.02)
1. Adjust. longitudinal WC	ref	0.02 (-0.01,0.05)	0.02 (-0.02,0.05)	0.02 (-0.01,0.06)	0.25	0.00 (-0.01,0.01)	0.00 (-0.02,0.01)	0.02 (0.00,0.04)**	0.00 (-0.02,0.02)
2. Adjust. CVD risk factors	ref	0.04 (0.01,0.08)	0.04 (0.01,0.08)	0.05 (0.02,0.09)	0.01*	0.01 (0.00,0.02)	0.00 (-0.02,0.02)	0.03 (0.01,0.06)**	0.00 (-0.01,0.02)
3. Adjust. other dairy intake	ref	0.03 (-0.01,0.07)	0.04 (0.00,0.07)	0.05 (0.01,0.09)	0.02*	0.01 (0.00,0.02)	0.00 (-0.02,0.02)	0.03 (0.01,0.06)**	0.00 (-0.01,0.02)
4. Exclusion CHD	ref	0.03 (-0.01,0.07)	0.04 (0.00,0.08)	0.06 (0.02,0.10)	0.004*	0.02 (0.00,0.03)*	0.01 (-0.01,0.03)	0.04 (0.01,0.06)**	0.01 (-0.01,0.03)
5. Energy adjusted dairy	ref	0.01 (-0.03,0.05)	0.04 (0.00,0.07)	0.04 (0.00,0.08)	0,01*	0.01 (0.00,0.02)	0.00 (-0.01,0.02)	0.03 (0.01,0.06)*	0.01 (-0.01,0.02)
**High-fat milk**									
Main analysis model 2	ref	0.00 (-0.04,0.04)	-0.03 (-0.06,0.01)	0.00 (-0.04,0.03)	0.46	-0.02 (-0.04,0.00)	-0.01 (-0.04,0.01)	-0.02 (-0.08,0.04)	-0.02 (-0.05,0.00)

1. Adjust. longitudinal WC	ref	0.02 (-0.01,0.06)	0.00 (-0.04,0.03)	0.01 (-0.02,0.04)	0.95	-0.01 (-0.03,0.01)	0.00 (-0.04,0.03)	-0.02 (-0.06,0.03)	-0.01 (-0.03,0.01)
2. Adjust. CVD risk factors	ref	0.01 (-0.03,0.04)	-0.01 (-0.05,0.02)	0.01 (-0.02,0.05)	0.77	-0.01 (-0.03,0.00)	-0.01 (-0.04,0.02)	-0.01 (-0.06,0.04)	-0.01 (-0.04,0.01)
3. Adjust. other dairy intake	ref	0.00 (-0.04,0.04)	-0.02 (-0.06,0.01)	0.00 (-0.04,0.04)	0.65	-0.02 (-0.04,0.00)	-0.01 (-0.05,0.02)	0.00 (-0.06,0.05)	-0.02 (-0.05,0.01)
4. Exclusion CHD	ref	-0.01 (-0.06,0.03)	-0.04 (-0.08,0.00)	-0.01 (-0.05,0.03)	0.47	-0.01 (-0.03,0.01)	0.00 (-0.04,0.04)	-0.01 (-0.07,0.05)	-0.02 (-0.05,0.01)
5. Energy adjusted dairy	ref	-0.03 (-0.07,0.02)	-0.04 (-0.08,0.00)	0.01 (-0.03,0.05)	0.85	-0.02 (-0.04,0.00)	-0.01 (-0.05,0.02)	-0.02 (-0.08,0.04)	-0.02 (-0.05,0.00)
**Low-fat milk**									
Main analysis model 2	ref	0.02 (-0.02,0.06)	0.02 (-0.02,0.06)	0.07 (0.03,0.11)	0.001**	0.02 (0.01,0.04)**	0.00 (-0.01,0.02)	0.04 (0.01,0.06)**	0.04 (0.01,0.06)**
1. Adjust. longitudinal WC	ref	0.01 (-0.02,0.04)	0.01 (-0.03,0.04)	0.03 (0.00,0.06)	0.15	0.01 (0.00,0.02)	0.00 (-0.02,0.02)	0.02 (0.00,0.04)*	0.01 (-0.01,0.04)
2. Adjust. CVD risk factors	ref	0.02 (-0.02,0.05)	0.02 (-0.01,0.05)	0.06 (0.02,0.09)	0.002**	0.02 (0.01,0.03)**	0.01 (-0.01,0.02)	0.04 (0.01,0.06)**	0.02 (0.00,0.05)
3. Adjust. other dairy intake	ref	0.02 (-0.02,0.06)	0.02 (-0.02,0.06)	0.07 (0.03,0.10)	0.002**	0.02 (0.01,0.04)**	0.01 (-0.01,0.03)	0.04 (0.01,0.06)**	0.03 (0.01,0.06)**
4. Exclusion CHD	ref	0.03 (-0.01,0.07)	0.03 (-0.01,0.07)	0.08 (0.04,0.12)	0.0004***	0.03 (0.01,0.04)***	0.01 (-0.01,0.03)	0.04 (0.01,0.07)**	0.04 (0.01,0.07)
5. Energy adjusted dairy	ref	0.03 (-0.01,0.06)	0.03 (-0.01,0.06)	0.08 (0.04,0.11)	0.0002***	0.02 (0.01,0.04)***	0.01 (-0.01,0.03)	0.04 (0.01,0.06)**	0.04 (0.01,0.06)*
**Total yogurt**									
Main analysis model 2	ref	0.04 (0.01,0.08)	0.00 (-0.04,0.04)	-0.01 (-0.05,0.02)	0.18	-0.02 (-0.04,0.00)	-0.01 (-0.04,0.03)	-0.01 (-0.06,0.05)	-0.03 (-0.06,0.00)
1. Adjust. longitudinal WC	ref	0.03 (0.00,0.06)	0.00 (-0.03,0.04)	-0.02 (-0.05,0.02)	0.14	-0.02 (-0.04,0.00)	-0.01 (-0.05,0.03)	-0.01 (-0.05,0.04)	-0.02 (-0.05,0.00)
2. Adjust. CVD risk factors	ref	0.03 (0.00,0.07)	0.00 (-0.03,0.04)	-0.02 (-0.05,0.01)	0.12	-0.02 (-0.04,0.00)	-0.01 (-0.05,0.03)	-0.01 (-0.06,0.04)	-0.03 (-0.06,0.00)*
3. Adjust. other dairy intake	ref	0.04 (0.01,0.08)	0.00 (-0.04,0.04)	-0.01 (-0.05,0.03)	0.23	-0.02 (-0.04,0.01)	0.00 (-0.05,0.04)	0.00 (-0.06,0.05)	-0.03 (-0.06,0.00)
4. Exclusion CHD	ref	0.05 (0.01,0.09)	0.01 (-0.03,0.06)	-0.01 (-0.05,0.03)	0.27	-0.02 (-0.04,0.01)	0.00 (-0.05,0.04)	0.02 (-0.04,0.08)	-0.03 (-0.06,0.00)
5. Energy adjusted dairy	ref	0.03 (0.00,0.07)	0.01 (-0.03,0.04)	-0.02 (-0.05,0.02)	0.12	-0.02 (-0.04,0.00)	-0.01 (-0.05,0.04)	-0.01 (-0.06,0.04)	-0.03 (-0.06,0.00)
**High-fat yogurt**									
Main analysis model 2	ref	-0.01 (-0.06,0.04)	0.00 (-0.05,0.05)	-0.10 (-0.16,-0.05)	0.0003***	-0.08 (-0.13,-0.03)**	0.01 (-0.01,0.02)	-0.11 (-0.25,0.03)	-0.11 (-0.18,-0.03)**
1. Adjust. longitudinal WC	ref	0.01 (-0.03,0.05)	0.02 (-0.02,0.06)	-0.06 (-0.10,-0.01)	0.04*	-0.04 (-0.08,0.01)	-0.02 (-0.09,0.04)	-0.05 (-0.17,0.07)	-0.05 (-0.11,0.02)
2. Adjust. CVD risk factors	ref	-0.01 (-0.06,0.03)	-0.01 (-0.06,0.03)	-0.07 (-0.12,-0.02)	0.005**	-0.05 (-0.10,-0.01)*	-0.02 (-0.08,0.05)	-0.09 (-0.22,0.05)	-0.08 (-0.15,-0.01)*
3. Adjust. other dairy intake	ref	-0.01 (-0.06,0.04)	0.00 (-0.05,0.05)	-0.10 (-0.16,-0.05)	0.0004***	-0.07 (-0.12,-0.03)**	-0.04 (-0.11,0.03)	-0.10 (-0.24,0.04)	-0.11 (-0.18,-0.03)*
4. Exclusion CHD	ref	-0.01 (-0.06,0.04)	-0.02 (-0.07,0.03)	-0.11 (-0.17,-0.05)	0.0009***	-0.07 (-0.12,-0.02)**	-0.04 (-0.11,0.04)	-0.09 (-0.24,0.07)	-0.11 (-0.19,-0.03)*
5. Energy adjusted dairy	ref	-0.03 (-0.08,0.02)	0.03 (-0.02,0.08)	-0.11 (-0.16,-0.06)	0.0001***	-0.08 (-0.13,-0.03)**	-0.04 (-0.11,0.03)	-0.11 (-0.25,0.03)	-0.11 (-0.19,-0.03)*
**Low-fat yogurt**									
Main analysis model 2	ref	0.03 (0.00,0.07)	0.07 (0.03,0.10)	0.01 (-0.02,0.05)	0.52	0.00 (-0.03,0.02)	-0.02 (-0.06,0.03)	0.01 (-0.05,0.06)	-0.01 (-0.04,0.02)
1. Adjust. longitudinal WC	ref	0.02 (-0.01,0.05)	0.05 (0.02,0.08)	0.00 (-0.03,0.03)	0.77	-0.01 (-0.03,0.01)	0.00 (-0.05,0.04)	0.00 (-0.05,0.04)	-0.02 (-0.04,0.01)
2. Adjust. CVD risk factors	ref	0.03 (-0.01,0.06)	0.05 (0.01,0.08)	0.00 (-0.03,0.03)	0.94	-0.01 (-0.03,0.01)	-0.01 (-0.05,0.04)	0.00 (-0.05,0.05)	-0.02 (-0.05,0.01)
3. Adjust. other dairy intake	ref	0.03 (0.00,0.07)	0.07 (0.03,0.10)	0.02 (-0.02,0.05)	0.47	0.00 (-0.03,0.02)	0.01 (-0.04,0.06)	0.01 (-0.04,0.07)	-0.01 (-0.04,0.02)
4. Exclusion CHD	ref	0.04 (0.00,0.08)	0.07 (0.03,0.11)	0.02 (-0.02,0.06)	0.47	0.00 (-0.02,0.02)	0.01 (-0.04,0.07)	0.04 (-0.02,0.10)	-0.01 (-0.05,0.02)
5. Energy adjusted dairy	ref	0.04 (0.00,0.07)	0.06 (0.02,0.09)	0.02 (-0.02,0.05)	0.41	0.00 (-0.03,0.02)	0.01 (-0.04,0.06)	0.00 (-0.05,0.06)	-0.01 (-0.04,0.02)
**Total cheese**									
Main analysis model 2	ref	0.02 (-0.02,0.06)	0.00 (-0.04,0.04)	0.01 (-0.03,0.05)	0.62	0.00 (-0.01,0.01)	-0.05 (-0.12,0.02)	0.00 (-0.02,0.01)	0.01 (-0.01,0.02)
1. Adjust. longitudinal WC	ref	0.00 (-0.03,0.03)	-0.02 (-0.05,0.01)	-0.01 (-0.05,0.02)	0.40	0.00 (-0.01,0.00)	-0.01 (-0.03,0.00)	0.00 (-0.01,0.01)	0.00 (-0.01,0.02)
2. Adjust. CVD risk factors	ref	0.03 (-0.01,0.06)	0.01 (-0.03,0.04)	0.01 (-0.03,0.04)	0.98	0.00 (-0.01,0.01)	0.00 (-0.02,0.02)	-0.01 (-0.02,0.01)	0.01 (-0.01,0.02)
3. Adjust. other dairy intake	ref	0.02 (-0.02,0.06)	0.00 (-0.04,0.04)	0.02 (-0.03,0.07)	0.31	0.01 (0.00,0.02)	0.01 (-0.02,0.03)	0.02 (0.00,0.05)	0.00 (-0.02,0.03)
4. Exclusion CHD	ref	0.02 (-0.02,0.06)	-0.01 (-0.05,0.04)	0.01 (-0.03,0.05)	0.69	0.00 (-0.01,0.01)	0.00 (-0.02,0.02)	0.00 (-0.01,0.01)	0.00 (-0.01,0.02)
5. Energy adjusted dairy	ref	0.01 (-0.02,0.05)	0.00 (-0.04,0.04)	0.01 (-0.03,0.05)	0.53	0.00 (-0.01,0.01)	0.00 (-0.02,0.02)	0.00 (-0.02,0.01)	0.01 (-0.01,0.02)
**High-fat cheese**									
Main analysis model 2	ref	0.01 (-0.02,0.05)	0.00 (-0.04,0.04)	0.00 (-0.04,0.04)	0.92	0.00 (-0.01,0.01)	0.00 (-0.05,0.05)	0.00 (-0.01,0.01)	0.00 (-0.02,0.02)
1. Adjust. longitudinal WC	ref	0.02 (-0.01,0.05)	0.01 (-0.03,0.04)	0.01 (-0.03,0.04)	0.90	0.00 (-0.01,0.01)	-0.02 (-0.03,0.00)	0.00 (-0.01,0.01)	0.01 (-0.01,0.03)
2. Adjust. CVD risk factors	ref	0.02 (-0.01,0.06)	0.02 (-0.02,0.05)	0.00 (-0.03,0.04)	0.92	0.00 (-0.01,0.01)	0.00 (-0.02,0.02)	0.00 (-0.02,0.01)	0.00 (-0.01,0.02)
3. Adjust. other dairy intake	ref	0.02 (-0.02,0.05)	0.00 (-0.04,0.04)	0.01 (-0.04,0.05)	0.56	0.01 (-0.01,0.02)	0.00 (-0.02,0.03)	0.02 (0.00,0.05)	0.00 (-0.03,0.02)
4. Exclusion CHD	ref	0.02 (-0.02,0.06)	-0.01 (-0.05,0.03)	-0.01 (-0.05,0.04)	0.72	0.00 (-0.01,0.01)	-0.01 (-0.03,0.01)	0.00 (-0.01,0.02)	0.00 (-0.02,0.02)
5. Energy adjusted dairy	ref	0.00 (-0.04,0.04)	-0.01 (-0.05,0.03)	-0.01 (-0.05,0.03)	0.96	0.00 (-0.01,0.01)	0.00 (-0.02,0.02)	0.00 (-0.01,0.01)	0.00 (-0.02,0.02)
**Low-fat cheese**									
Main analysis model 2	ref	0.02 (-0.02,0.07)	0.01 (-0.04,0.06)	0.02 (-0.03,0.07)	0.50	0.01 (-0.01,0.03)	0.00 (-0.02,0.02)	-0.01 (-0.06,0.03)	0.02 (-0.01,0.05)
1. Adjust. longitudinal WC	ref	0.02 (-0.02,0.06)	-0.03 (-0.07,0.01)	-0.03 (-0.07,0.01)	0.09	-0.01 (-0.03,0.01)	0.02 (-0.02,0.05)	-0.03 (-0.07,0.00)	-0.01 (-0.04,0.01)
2. Adjust. CVD risk factors	ref	0.00 (-0.04,0.04)	-0.01 (-0.05,0.04)	-0.01 (-0.05,0.03)	0.46	0.00 (-0.02,0.02)	-0.01 (-0.04,0.03)	-0.03 (-0.07,0.01)	0.01 (-0.02,0.04)
3. Adjust. other dairy intake	ref	0.02 (-0.02,0.07)	0.02 (-0.03,0.06)	0.02 (-0.03,0.08)	0.35	0.02 (-0.01,0.04)	0.02 (-0.02,0.06)	0.01 (-0.04,0.06)	0.02 (-0.02,0.05)
4. Exclusion CHD	ref	0.01 (-0.03,0.06)	0.02 (-0.03,0.07)	0.02 (-0.03,0.07)	0.46	0.01 (-0.01,0.03)	0.03 (-0.02,0.07)	0.00 (-0.05,0.04)	0.01 (-0.02,0.04)
5. Energy adjusted dairy	ref	0.02 (-0.03,0.07)	0.01 (-0.04,0.06)	0.02 (-0.03,0.07)	0.49	0.01 (-0.01,0.03)	0.02 (-0.02,0.06)	-0.01 (-0.06,0.03)	0.02 (-0.01,0.05)
**Cream**									
Main analysis model 2	ref	-0.03 (-0.07,0.02)	-0.05 (-0.09,0.00)	-0.07 (-0.12,-0.03)	0.77	-0.02 (-0.05,0.01)	0.00 (-0.02,0.01)	-0.13 (-0.26,0.00)	-0.10 (-0.19,-0.02)*
1. Adjust. longitudinal WC	ref	-0.01 (-0.05,0.02)	-0.03 (-0.07,0.01)	-0.03 (-0.07,0.01)	0.62	-0.01 (-0.03,0.02)	0.00 (-0.02,0.03)	-0.04 (-0.16,0.07)	-0.05 (-0.12,0.02)*

2. Adjust. CVD risk factors	ref	-0.01 (-0.05,0.03)	-0.03 (-0.07,0.01)	-0.03 (-0.07,0.01)	0.67	-0.01 (-0.03,0.02)	0.00 (-0.02,0.03)	-0.11 (-0.23,0.02)	-0.03 (-0.11,0.04)
3. Adjust. other dairy intake	ref	-0.03 (-0.07,0.02)	-0.05 (-0.09,0.00)	-0.07 (-0.12,-0.02)	0.94	-0.02 (-0.05,0.01)	0.00 (-0.03,0.03)	-0.10 (-0.24,0.03)	-0.11 (-0.20,-0.02)*
4. Exclusion CHD	ref	-0.03 (-0.07,0.02)	-0.05 (-0.10,0.00)	-0.08 (-0.13,-0.03)	0.93	-0.02 (-0.05,0.01)	0.00 (-0.03,0.04)	-0.13 (-0.27,0.01)	-0.11 (-0.20,-0.02)*
5. Energy adjusted dairy	ref	-0.04 (-0.09,0.00)	-0.01 (-0.06,0.03)	-0.08 (-0.13,-0.04)	0.51	-0.02 (-0.05,0.01)	0.00 (-0.03,0.03)	-0.12 (-0.26,0.01)	-0.10 (-0.19,-0.01)*
**Ice cream**									
Main analysis model 2	ref	0.03 (-0.02,0.07)	0.02 (-0.02,0.07)	0.01 (-0.03,0.05)	0.52	0.04 (-0.01,0.08)	0.01 (-0.03,0.05)	0.06 (0.00,0.11)*	-0.02 (-0.12,0.08)
1. Adjust. longitudinal WC	ref	0.02 (-0.01,0.06)	0.02 (-0.02,0.06)	0.00 (-0.03,0.04)	0.72	0.00 (-0.04,0.04)	-0.05 (-0.21,0.11)	0.01 (-0.04,0.05)	-0.01 (-0.09,0.08)
2. Adjust. CVD risk factors	ref	0.03 (-0.01,0.07)	0.04 (0.00,0.08)	0.02 (-0.02,0.05)	0.18	0.04 (0.00,0.08)	-0.05 (-0.21,0.12)	0.06 (0.01,0.11)*	-0.01 (-0.10,0.08)
3. Adjust. other dairy intake	ref	0.03 (-0.02,0.07)	0.02 (-0.02,0.07)	0.01 (-0.03,0.05)	0.42	0.05 (0.00,0.10)*	-0.04 (-0.21,0.14)	0.08 (0.03,0.14)**	-0.02 (-0.13,0.08)
4. Exclusion CHD	ref	0.02 (-0.02,0.07)	0.03 (-0.02,0.07)	0.01 (-0.03,0.05)	0.47	0.04 (-0.01,0.09)	-0.04 (-0.24,0.15)	0.06 (0.01,0.11)*	-0.01 (-0.11,0.09)
5. Energy adjusted dairy	ref	0.05 (0.01,0.09)	0.00 (-0.04,0.04)	0.00 (-0.04,0.05)	0.58	0.03 (-0.01,0.08)	-0.03 (-0.20,0.13)	0.06 (0.00,0.11)*	-0.02 (-0.12,0.08)

Model 2 included age (continuous), sex and energy intake (continuous), education (3 categories), smoking (3 categories), physical activity (continuous), alcohol consumption (4 categories), family history of diabetes (yes/no, RS-I and RS-II only) and intakes of fruit, vegetables, wholegrains, legumes, nuts, tea, coffee, red meat and sugar-sweetened beverages (continuous). CVD, Cardiovascular Disease; CHD, Coronary Heart Disease; WC, Waist Circumference. P value significance level: *.05, **.01, ***.001

Repeated measures of dairy intake

Table S12. Mean dairy intake at baseline and follow-up in a subsample of RS-I and RS-II (*n*=1887).

	RS-I n=1,028		RS-II n=859	
	Baseline	Follow-up	Baseline	Follow-up
**Total dairy**	3.85 ± 1.69	4.21 ± 2.54	2.93 ± 1.80	2.83 ± 1.70
**High-fat dairy**	2.12 ± 1.35	2.45 ± 2.23	1.40 ± 1.40	1.37 ± 1.38
**Low-fat dairy**	1.72 ± 1.40	1.68 ± 1.49	1.53 ± 1.36	1.46 ± 1.17
**Total fermented dairy**	2.70 ± 1.43	3.10 ± 2.30	1.98 ± 1.50	2.02 ± 1.43
**High-fat fermented dairy**	1.77 ± 1.18	2.20 ± 2.17	1.07 ± 1.18	1.11 ± 1.20
**Low-fat fermented dairy**	0.93 ± 1.10	0.91 ± 1.11	0.92 ± 1.09	0.92 ± 0.99
**Total milk**	1.43 ± 1.09	1.28 ± 1.20	1.00 ± 1.04	0.87 ± 0.91
**High-fat milk**	0.23 ± 0.55	0.21 ± 0.50	0.25 ± 0.70	0.19 ± 0.55
**Low-fat milk**	1.20 ± 1.09	1.07 ± 1.15	0.75 ± 0.80	0.68 ± 0.71
**Total yogurt**	0.47 ± 0.49	0.36 ± 0.52	0.61 ± 0.78	0.59 ± 0.72
**High-fat yogurt**	0.14 ± 0.29	0.03 ± 0.16	0.11 ± 0.42	0.08 ± 0.25
**Low-fat yogurt**	0.33 ± 0.42	0.32 ± 0.52	0.50 ± 0.71	0.51 ± 0.71
**Total cheese**	1.88 ± 1.11	2.47 ± 2.13	1.34 ± 1.25	1.41 ± 1.21
**High-fat cheese**	1.66 ± 1.15	2.16 ± 2.16	0.95 ± 1.11	1.02 ± 1.17
**Low-fat cheese**	0.22 ± 0.61	0.31 ± 0.70	0.39 ± 0.82	0.39 ± 0.66
**Cream**	0.26 ± 0.69	0.06 ± 0.20	0.12 ± 0.21	0.11 ± 0.21
**Ice cream**	0.04 ± 0.11	0.12 ± 0.37	0.15 ± 0.35	0.15 ± 0.25

Table S13. Associations of dairy product types and prediabetes risk using repeated measures of dairy intakes as time-dependent exposure (*n*=6053) or adjusting for dairy intake at follow-up (*n*=1707).

	**RS-I** Continuous HR (95%CI)	**RS-II** Continuous HR (95%CI)
**Total dairy**		
Model 2	1.08 (1.03-1.14)**	1.03 (0.98-1.08)
Time-dependent exposure	1.08 (1.02-1.14)**	1.01 (0.96-1.07)
Adjusted for FU intake	1.13 (1.04-1.22)**	1.01 (0.94-1.08)
**High-fat dairy**		
Model 2	1.04 (0.98-1.12)	1.00 (0.95-1.06)
Time-dependent exposure	1.04 (0.98-1.12)	0.99 (0.93-1.05)
Adjusted for FU intake	1.09 (0.99-1.20)	0.99 (0.92-1.07)
**Low-fat dairy**		
Model 2	1.07 (1.01-1.14)*	1.06 (0.99-1.14)
Time-dependent exposure	1.07 (1.01-1.14)*	1.06 (0.99-1.14)
Adjusted for FU intake	1.08 (0.98-1.18)*	1.04 (0.94-1.15)
**Total fermented**		
Model 2	1.06 (1.00-1.12)*	1.02 (0.97-1.07)
Time-dependent exposure	1.06 (1.00-1.12)*	1.00 (0.95-1.05)
Adjusted for FU intake	1.10 (1.01-1.20)	1.01 (0.95-1.09)
**High-fat fermented**		
Model 2	1.07 (1.00-1.15)	1.02 (0.96-1.07)
Time-dependent exposure	1.07 (1.00-1.15)	0.99 (0.94-1.05)
Adjusted for FU intake	1.09 (0.98-1.22)	1.01 (0.94-1.09)
**Low-fat fermented**		
Model 2	1.03 (0.94-1.12)	1.01 (0.91-1.11)
Time-dependent exposure	1.03 (0.94-1.12)	1.01 (0.92-1.11)
Adjusted for FU intake	1.07 (0.95-1.20)	1.02 (0.89-1.18)
**Total milk**		
Model 2	1.06 (0.99-1.15)	1.05 (0.95-1.16)
Time-dependent exposure	1.06 (0.99-1.15)	1.07 (0.97-1.19)
Adjusted for FU intake	1.08 (0.96-1.21)	0.94 (0.82-1.09)
**High-fat milk**		
Model 2	0.95 (0.81-1.10)	0.62 (0.43-0.91)*

Time-dependent exposure	0.95 (0.81-1.10)	0.88 (0.67-1.15)
Adjusted for FU intake	1.07 (0.89-1.28)	0.56 (0.34-0.91)*
**Low-fat milk**		
Model 2	1.08 (1.00-1.16)	**1.11 (1.00-1.23)***
Time-dependent exposure	1.08 (1.00-1.16)	1.11 (1.00-1.23)
Adjusted for FU intake	1.04 (0.92-1.17)	1.00 (0.87-1.15)
**Total yogurt**		
Model 2	0.98 (0.80-1.20)	0.91 (0.68-1.22)
Time-dependent exposure	0.89 (0.73-1.07)	1.03 (0.84-1.25)
Adjusted for FU intake	0.91 (0.69-1.21)	1.06 (0.79-1.41)
**High-fat yogurt**		
Model 2	0.76 (0.54-1.07)	0.47 (0.20-1.10)
Time-dependent exposure	0.76 (0.54-1.07)	0.66 (0.34-1.30)
Adjusted for FU intake	0.71 (0.44-1.15)	0.30 (0.08-1.21)
**Low-fat yogurt**		
Model 2	0.97 (0.78-1.21)	1.03 (0.82-1.29)
Time-dependent exposure	0.97 (0.78-1.21)	1.07 (0.89-1.30)
Adjusted for FU intake	1.02 (0.74-1.41)	1.15 (0.87-1.51)
**Total cheese**		
Model 2	1.11 (1.04-1.19)**	1.02 (0.97-1.08)
Time-dependent exposure	1.11 (1.04-1.19)**	1.00 (0.94-1.05)
Adjusted for FU intake	1.19 (1.06-1.33)**	1.01 (0.94-1.09)
**High-fat cheese**		
Model 2	1.08 (1.01-1.16)*	1.02 (0.97-1.08)
Time-dependent exposure	1.08 (1.01-1.16)*	1.00 (0.94-1.06)
Adjusted for FU intake	1.11 (0.99-1.24)*	1.01 (0.94-1.09)
**Low-fat cheese**		
Model 2	1.11 (0.97-1.27)	1.00 (0.88-1.14)
Time-dependent exposure	1.13 (1.00-1.28)	1.07 (0.90-1.28)
Adjusted for FU intake	1.18 (0.99-1.41)	1.03 (0.83-1.27)
**Cream**		
Model 2	1.05 (0.93-1.18)	0.89 (0.50-1.59)
Time-dependent exposure	1.05 (0.93-1.18)	0.86 (0.48-1.55)
Adjusted for FU intake	1.05 (0.89-1.24)	0.85 (0.44-1.64)
**Ice cream**		
Model 2	0.75 (0.34-1.65)	0.96 (0.65-1.43)
Time-dependent exposure	0.75 (0.34-1.65)	1.05 (0.72-1.53)
Adjusted for FU intake	1.25 (0.38-4.05)	0.91 (0.56-1.49)

Model 2 included age (continuous), sex, energy intake (continuous), education (3 categories), smoking (3 categories), physical activity (continuous), alcohol consumption (4 categories), family history of diabetes (yes/no) and fruits, vegetables, wholegrains, legumes, nuts, tea, coffee, red meat and sugar-sweetened beverages (SSB) intakes (continuous). HR, Hazard Ratio. P value significance level: *.05, **.01, ***.001

Table S14. Associations of dairy product intakes and longitudinal insulin resistance with repeated measures of dairy intake as fixed effect in RS-I and RS-II (*n*=4274).

	**RS-I**<br>n=1021<br>Continuous<br>B (95%CI)	**RS-II**<br>n=857<br>Continuous<br>B (95%CI)
**Total dairy**		
Model 2	0.00 (-0.01,0.02)	0.01 (-0.01,0.02)
Repeated measures	-0.01 (-0.02, 0.00)	0.00 (-0.01, 0.01)
**High-fat dairy**		
Model 2	-0.01 (-0.02,0.01)	0.00 (-0.02,0.01)
Repeated measures	-0.01 (-0.02, 0.01)	0.00 (-0.01, 0.01)
**Low-fat dairy**		
Model 2	0.01 (0.00,0.03)	0.02 (0.00,0.04)*
Repeated measures	0.00 (-0.01, 0.01)	0.01 (-0.01, 0.02)

**Total fermented**		
Model 2	0.00 (-0.02,0.01)	0.00 (-0.02,0.01)
Repeated measures	-0.01 (-0.02, 0.01)	0.00 (-0.01, 0.01)
**High-fat fermented**		
Model 2	0.00 (-0.02,0.01)	0.00 (-0.02,0.01)
Repeated measures	0.00 (-0.02, 0.01)	0.00 (-0.02, 0.01)
**Low-fat fermented**		
Model 2	0.00 (-0.02,0.02)	0.00 (-0.03,0.02)
Repeated measures	-0.01 (-0.03, 0.01)	0.00 (-0.02, 0.02)
**Total milk**		
Model 2	0.00 (-0.02,0.02)	0.03 (0.01,0.06)**
Repeated measures	-0.01 (-0.02, 0.01)	0.01 (-0.01, 0.03)
**High-fat milk**		
Model 2	-0.02 (-0.05,0.02)	-0.02 (-0.08,0.04)
Repeated measures	-0.02 (-0.05, 0.01)	0.00 (-0.04, 0.05)
**Low-fat milk**		
Model 2	0.01 (-0.01,0.03)	0.04 (0.01,0.06)**
Repeated measures	0.00 (-0.02, 0.01)	0.02 (-0.01, 0.04)
**Total yogurt**		
Model 2	-0.01 (-0.05,0.04)	-0.01 (-0.06,0.05)
Repeated measures	-0.03 (-0.06, 0.00)	0.02 (-0.01, 0.06)
**High-fat yogurt**		
Model 2	-0.04 (-0.11,0.03)	-0.11 (-0.25,0.03)
Repeated measures	-0.07 (-0.12, -0.02)**	0.04 (-0.05, 0.13)
**Low-fat yogurt**		
Model 2	0.01 (-0.04,0.06)	0.01 (-0.05,0.06)
Repeated measures	-0.02 (-0.05, 0.02)	0.02 (-0.02, 0.07)
**Total cheese**		
Model 2	0.00 (-0.02,0.02)	0.00 (-0.02,0.01)
Repeated measures	0.00 (-0.01, 0.02)	-0.01 (-0.02, 0.00)
**High-fat cheese**		
Model 2	0.00 (-0.02,0.02)	0.00 (-0.01,0.01)
Repeated measures	0.00 (-0.01, 0.02)	-0.01 (-0.02, 0.00)
**Low-fat cheese**		
Model 2	0.02 (-0.02,0.06)	-0.01 (-0.06,0.03)
Repeated measures	0.00 (-0.03, 0.02)	-0.02 (-0.05, 0.01)
**Cream**		
Model 2	0.00 (-0.03,0.03)	-0.13 (-0.26,0.00)
Repeated measures	0.01 (-0.02, 0.04)	-0.02 (-0.12, 0.07)
**Ice cream**		
Model 2	-0.04 (-0.21,0.13)	0.06 (0.00,0.11)*
Repeated measures	0.06 (-0.02, 0.13)	0.04 (0.00, 0.09)

Model 2 included age (continuous), sex, energy intake (continuous), education (3 categories), smoking (3 categories), physical activity (continuous), alcohol consumption (4 categories), family history of diabetes (yes/no) and fruits, vegetables, wholegrains, legumes, nuts, tea, coffee, red meat and sugar-sweetened beverages (SSB) intakes (continuous). P value significance level: *.05, **.01, ***.001
